# Encapsulation of Bioactive Phytochemicals in Plant-Based Matrices and Application as Additives in Meat and Meat Products

**DOI:** 10.3390/molecules26133984

**Published:** 2021-06-29

**Authors:** Rubén Domínguez, Mirian Pateiro, Paulo E. S. Munekata, David Julian McClements, José M. Lorenzo

**Affiliations:** 1 Centro Tecnológico de la Carne de Galicia, Rúa Galicia No. 4, Parque Tecnológico de Galicia, San Cibrao das Viñas, 32900 Ourense, Spain; rubendominguez@ceteca.net (R.D.); mirianpateiro@ceteca.net (M.P.); paulosichetti@ceteca.net (P.E.S.M.); jmlorenzo@ceteca.net (J.M.L.); 2 Department of Food Science, University of Massachusetts Amherst, 100 Holdsworth Way, Amherst, MA 01003, USA; 3 Área de Tecnología de los Alimentos, Facultad de Ciencias de Ourense, Universidad de Vigo, 32004 Ourense, Spain

**Keywords:** plant-based foods, encapsulation, delivery systems, nutraceuticals, bioactive ingredients, botanical extracts

## Abstract

The development of plant-based functional food ingredients has become a major focus of the modern food industry as a response to changes in consumer attitudes. In particular, many consumers are switching to a plant-based diet because of their concerns about animal-derived foods on the environment, human health, and animal welfare. There has therefore been great interest in identifying, isolating, and characterizing functional ingredients from botanical sources, especially waste streams from food and agricultural production. However, many of these functional ingredients cannot simply be incorporated into foods because of their poor solubility, stability, or activity characteristics. In this article, we begin by reviewing conventional and emerging methods of extracting plant-based bioactive agents from natural resources including ultrasound-, microwave-, pulsed electric field- and supercritical fluid-based methods. We then provide a brief overview of different methods to characterize these plant-derived ingredients, including conventional, chromatographic, spectroscopic, and mass spectrometry methods. Finally, we discuss the design of plant-based delivery systems to encapsulate, protect, and deliver these functional ingredients, including micelles, liposomes, emulsions, solid lipid nanoparticles, and microgels. The potential benefits of these plant-based delivery systems are highlighted by discussing their use for incorporating functional ingredients into traditional meat products. However, the same technologies could also be employed to introduce functional ingredients into plant-based meat analogs.

## 1. Introduction

The food and agricultural industries have become increasingly interested in developing sustainable plant-based functional materials to replace synthetic or animal-based ones. This change in emphasis has been driven by growing consumer demands for a more ethical, healthy, and environmentally-friendly food supply to feed the expanding global population [1,2]. Scientists are therefore utilizing plant-derived constituents, such as proteins, polysaccharides, phospholipids, and lipids, to construct new materials for use in the food industry, including foods, beverages, packaging materials, coatings, and delivery systems [3,4,5,6]. In this article, we focus on the design, fabrication, and utilization of plant-based delivery systems for bioactive food ingredients, such as nutraceuticals, preservatives, colors, and flavors. As a specific example, we highlight the utility of these delivery systems for incorporating functional ingredients into traditional meat products. In recent decades, a great deal of research has focused on the isolation, characterization, and utilization of phytochemicals in the food industry [7,8,9,10]. As a specific example, they are being utilized as natural antioxidants and antimicrobials to inhibit chemical degradation and microbial growth in meat and meat products [11]. However, many plant-based bioactive agents cannot simply be applied to agricultural crops or incorporated into food products or packaging materials because of their poor solubility, their low chemical stability, or their adverse impacts on food quality (such as appearance, texture, or flavor). Consequently, it is important to encapsulate these components within colloidal particles that are specially designed to improve their water dispersibility, chemical stability, and matrix compatibility [4,5]. Moreover, encapsulation of these phytochemicals can improve food quality by masking off odors and flavors [12,13], while increasing their shelf-life. The particles may vary in their compositions, dimensions, shapes, electrical properties, and environmental sensitivities. As a result, it is important to select the most appropriate particle design for each specific application. As a representative example, we focus on the application of encapsulated phytochemicals for improving the quality, safety, and shelf-life of meat products in this manuscript.

This article reviews the main phytochemicals that can be used as functional additives in foods, highlights the various techniques that can be used to isolate them from botanical sources, and provides a brief overview of the different analytical instruments that can be used to establish their identity and concentration. Advanced encapsulation technologies that can be used to increase the handling, stability, and efficacy of phytochemicals are then discussed. Finally, the efficacy of encapsulated phytochemicals is highlighted by reviewing their potential applications in the meat industry.

## 2. Isolation of Bioactive Agents from Botanical Sources

**Figure 1:**
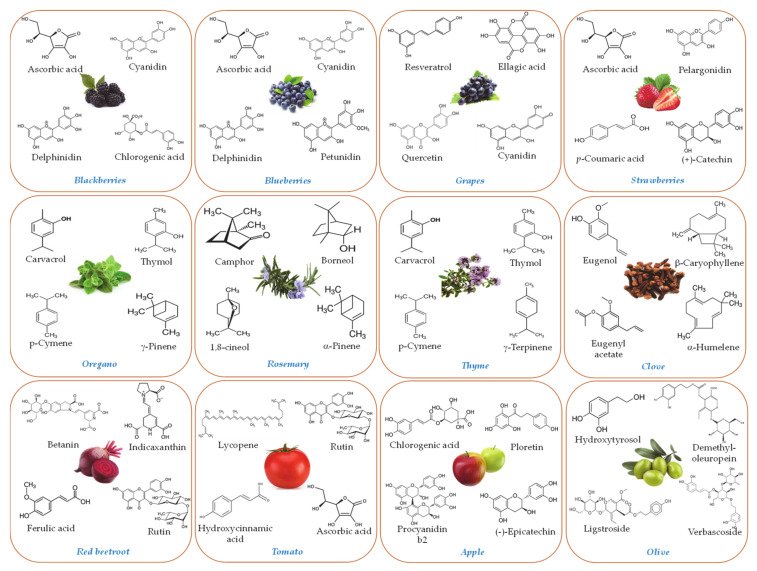
Examples of some important bioactive agents isolated from edible plant materials.

In the last two decades, there has been increasing interest in the extraction of bioactive compounds from plant matrices due to their nutritional value, technological properties, and potential health benefits [14]. In addition, the valorization of agro-food by-products based on the extraction of high-value molecules and the development of functional products can lead to the more environmentally sustainable use of these resources and higher economic benefits for the food sector [10,15]. In general, bioactive compounds can be subdivided into two main groups: *lipophilic compounds* such as essential oils, oleoresins, curcuminoids, and carotenoids that are extracted with more non-polar solvents; and *hydrophilic compounds* such as polyphenols that are extracted using more polar solvents. A broad spectrum of different kinds of bioactive constituents are found in different plant sources [16] (Figure 1). As a result, crude extracts often contain a mixture of different phytochemicals that may have different biological activities, which may be additive, synergistic, or antagonistic. As an example, hydrophobic extracts from botanical sources (such as tomatoes or turmeric) may contain a mixture of different hydrophobic phytochemicals, such as carotenoids (lutein, lycopene, α-carotene and β-carotene), xanthophylls (lutein, zeaxanthin, astaxanthin and canthaxanthin), fat-soluble vitamins and pro-vitamins (retinol and tocopherols), curcuminoids and alkaloids [10,17,18]. Similarly, essential oils isolated from different varieties of aromatic plants (such as *Asteraceae, Lamiaceae, Lauraceae, Myrtaceae, Rutaceae, Umbelliferae,* and *Zingiberaceae* families) contain a great variety of bioactive compounds, which can be grouped into two main groups; terpenoids and phenylpropanoids [8,19,20]. Similarly, hydrophilic extracts from plants may also contain many different components that different in their biological activities. For instance, there are numerous kinds of polyphenols in aqueous extracts [7,21], including flavonoids (rutin, naringenin, naringenin chalcone, kaempferol, rhamnetin, astragalin, rhamnocitrin, quercetin, catechin, gallocatechin, tanins, etc.) and phenolic acids (hydroxycinnamic, chlorogenic, rosmarinic, sinapic, p-coumaric, ferulic, syringic, vanillic, caffeic acids, etc.). Moreover, in berries (elderberry, blueberry, blackberry, blackcurrant, cloudberry, bearberry, strayberry, etc.) one of the largest groups of polyphenols are anthocyanins and anthocyanidins (malvidin, peonidin, petunidin, cyanidin-3-O-sambubioside, cyanidin-3-O-glucoside, cyanidin 3-(E)-p-coumaryl-sambubioside-5-glucoside, etc.) [14,22]. Many anthocyanins are widely used as natural pigments and antioxidants in foods. Similarly, betalains (such as betacyanins and betaxanthins) are also used as natural pigments and antioxidants [9,23,24,25].

To extract these compounds, it is vital to choose a suitable extraction method. Ideally, this method should minimize the processing steps, time, and energy consumption involved, while increasing the quality and yield of the extract, and ensuring the safety of the final product. There are some common factors that affect the efficiency of extraction in both conventional or emerging assisted extractions, such as the solid/solvent ratio, the solvent concentration, particle size and the use of flow or batch mode [26]. Moreover, the solvent, extraction technique, and operating conditions used strongly influence extraction efficiency and the phytochemical composition of the extract obtained [19]. With this in mind, below is a description of the main methods used to obtain phytochemicals.

### 2.1. Conventional Extraction Methods

Conventional extraction methods include traditional solid–liquid and liquid–liquid extraction procedures. Solid–liquid extraction is the most popular method, in which the plant material is mixed with an appropriate solvent or solvent mixture that solubilizes the required active compounds, while in liquid–liquid extractions, plant material is mixed with two immiscible solvents and, according to solvent polarity, the target compounds are recovered from each fraction [14]. In these methods, the phytochemicals are extracted from plants using different techniques such as Soxhlet, percolation, or maceration methods [26,27]. Although relatively simple to implement, traditional extraction methods have numerous limitations, such as long-processing times, low efficiency, non-selectivity, tedious procedures, difficulties in automation, the formation of emulsions, and/or the use of high amounts of potentially toxic solvents [28,29].

In the case of oleoresins (a mixture of waxes, fatty acids, essential oils, carotenoids, and other lyophilic compounds) obtained from some plants such tomato or tomato pomace, high amounts of organic solvents (hexane, chloroform, acetone, etc.) are normally used [10]. In general, a solvent mixture that includes a combination of polar and non-polar solvents is utilized to ensure optimum extraction of lipophilic compounds with different polarities. However, it is important to highlight that some solvents contain peroxides, which can react with the phytochemicals and reduce the quality and functionality properties of extracted oleoresins [10]. In contrast, to extract polyphenols and other more hydrophilic compounds, hydroalcoholic solutions are the most commonly used solvents [9,14,16,22].

Essential oils can also be extracted using solvents but due to their volatile nature and low solubility in water, distillation procedures tend to be more commonly used [19,30]. The conventional methods for the extraction of essential oils are steam distillation and the hydrodistillation of dried plant materials [8,31,32]. In these processes, high temperatures cause the release of essential oils in vapor form, which are subsequently condensed to obtain a liquid mixture of essential oil/water [32]. Essential oils are usually immiscible in water and so can easily be separated by decantation. The main difference between hydrodistillation and steam distillation is that in hydrodistillation, the plant material is boiled with water at high temperatures, while in steam distillation, direct contact with the boiling water is avoided by supporting the plant material within a grid (separate place), and the steam is passed through the material to extract the essential oils [19,30]. Both techniques are relatively simple, easily installed and do not require expensive material. However, there are some important drawbacks, such as the degradation or transformation of some compounds caused by overheating or photo-oxidation, the hydrolysis of esterified compounds sensitive to water [33], or low yields due to the boiling temperature of water being much lower than that of some essential oil constituents (150–300 °C). Additionally, it is important to highlight that steam distillation reduces the extraction time, artifacts, and losses of polar molecules in comparison with hydrodistillation [32].

### 2.2. Extractions Assisted by Emerging Technologies

Traditional extraction methods are often time-consuming, laborious, and environmentally damaging. As previously mentioned, the consumption of high amounts of sample, energy, time and the use of high volumes of toxic and dangerous organic solvents are the main drawbacks of these technologies [27]. Thus, in order to overcome some of these problems, in recent years, the use of alternative technologies has emerged [14].

In this regard, several innovative and emerging extraction technologies, such as ultrasound-assisted extraction (USAE), microwave-assisted extraction (MWAE), pulsed electric field-assisted extraction (PEFAE), supercritical fluid-assisted extraction (SFAE), infrared-assisted extraction (IAE), accelerated solvent extraction (ASE), enzyme-assisted extraction (EAE), high-voltage electrical discharge extraction (HVED), high hydrostatic pressure extraction (HHPE) and pressurized liquid extraction (PLE), have been identified as more green methods for the separation of high-added functional ingredients from plant materials [34]. Although several new technologies have been developed, we focus this section on the most commonly used techniques, namely USAE, MWAE, PEFAE, and SFAE. These emerging technologies have several advantages, including low solvent, energy, and time consumption, use of more eco-friendly solvents, higher extraction yields, and higher selectivity [29,35]. The advantages and drawbacks of the application of emerging technologies for the extraction of bioactive compounds from botanical sources are summarized in Table 1. Moreover, the use of deep eutectic systems in combination with these emerging technologies offers a greener alternative to common organic solvents [36]. Indeed, the use of deep eutectic systems for the extraction of phytochemicals has grown considerably in recent years due to their ease of preparation and use. Combining deep eutectic systems with these emerging technologies is particularly useful for the extraction of the two major phenolic families, phenolic acids and flavonoids, such as anthocyanins, flavones, and flavonols [36]. In addition, it must be taken into account that these techniques must be capable of being scalable at an industrial level [26]. This is not a trivial task and often the results obtained at the laboratory scale are not reproducible when the same technology and extraction parameters are applied at the pilot/industrial scale.

#### 2.2.1. Ultrasound-Assisted Extraction (USAE)

Among all emerging technologies to assist phytochemical extraction, USAE has been most widely applied. This fact is due to the cost-effectiveness of the equipment required [27], as well as the increase in mixing efficiency, decrease in extraction time, and reduction in energy [28,37,38]. Additionally, the USAE can be performed at relatively low temperatures, which protects thermolabile compounds from degradation or volatilization [39]. Similarly, USAE also been shown to improve extraction yields and composition in comparison to conventional techniques [35].

In general, ultrasonic methods are normally categorized according to the power levels and frequencies employed into low-intensity (100 kHz–10 MHz; <1 W/cm^2^) and high-intensity (20 kHz–100 MHz; >1 W/cm^2^) methods. For extraction of bioactive compounds, only high-intensity ultrasound methods are appropriate because they can modify the physical properties of the samples. In contrast, low-intensity methods are non-destructive techniques that are normally used to monitor the physical and chemical properties of foods [35].

Ultrasonic waves need a proper medium for their propagation, such as a fluid or solid capable of transmitting high-frequency pressure waves. The ability of high-intensity ultrasonic waves to disrupt materials is mainly the result of a phenomenon called cavitation. During USAE, the implosion of cavitation bubbles produces a rapid change in temperature (>5000 K) and pressure (100 MPa), which causes the disruption of the cells of plant materials, enhances the transfer of phytochemicals into the solvent (higher surface area for mass transfer), and improves the penetration of solvents into the sample matrix [27,35]. The improvement of solvent penetration is also known as the ultrasonic capillary effect, which increases the depth and speed of penetration of fluids into channels and pores under certain sonication conditions [37]. Furthermore, the cavitation bubbles also cause macro-turbulences, which enhance solute–solvent mixing. Although the favoring of the extraction of phytochemicals is mainly attributed to the disruption of cells, it is important to take into account that cavitation bubbles cause a series of other effects on plant material, including surface peeling, erosion, and particle breakdown. Thus, several mechanism (fragmentation, erosion, sonocapillary effect, sonoporation, and local shear stresses) are involved in the destruction and detexturation of plant structures [37]. It is essential to highlight that all of these phenomena, as well as the extraction efficiency, depend on sonication conditions (power, frequency, duration, and temperature), as well as other parameters that influence the mass transfer of solutes from plant matrices to solvents, such as particle size and extraction time [27].

Numerous bioactive phytochemicals, such as carotenoids, antioxidants, colorants, and essential oils, have been obtained with USAE techniques [26,42]. In fact, it is considered to be one of the best non-thermal technologies for green phytochemical extractions. Several studies have been demonstrated that USAE treatments can enhance phytochemical extraction. For instance, a recent study demonstrated that USAE treatment improved the extraction of both lipophilic and hydrophilic substances from turmeric, ginger and garlic, including vitamin C, total phenols, flavonoids, non-flavonoids and β-carotene [43]. Similarly, USAE has been successfully used for the extraction of antioxidant compounds from *Decatropis bicolor—*a medicinal plant from Central America [44]. In this study, the authors optimized the extraction conditions, such as extraction time, temperature, and sample size [44].

Additionally, this technique can be combined with others to enhance the extraction yield and extract properties, such as ultrasonic-microwave or ultrasonic-pulsed electric field [28]. This aspect will be discussed in Section 2.2.5.

#### 2.2.2. Microwave-Assisted Extraction (MWAE)

Another promising emerging technology used for the extraction of phytochemicals is MWAE. This technology has been widely used to obtain aromas, antioxidants, colorants and other important bioactive compounds from plant materials [26]. The main advantages of MWAE in comparison to conventional extraction methods are the reduction in extraction times (from several hours to several seconds or minutes), the reduction in the amount of solvent used, and an improvement in the extraction yields [27,28,45].

This technique is based on propagating electromagnetic waves (0.1 to 3 GHz) through plant materials [27]. The microwave energy acts as non-ionizing radiation causing the generation of thermal energy [34,38]. However, non-polar solvents (with low dielectric constants) are transparent to microwaves, thus they are not heated. In these cases, low amounts of polar solvent are typically added to the non-polar solvent used in the extraction process to increase its dielectric constant. Furthermore, the moisture within plant materials serves as a target for the microwave energy. Due to the increase in temperature and pressure caused by microwave energy, a series of effects are produced in the plant material. These include the evaporation of water, the swelling of cells, and the dehydration of cellulose in the cell walls, thereby leading to changes in the matrix structures such as the disruption of plant cells, which increases the mass transfer of phytochemicals into the solvent [27]. However, several parameters may influence MWAE, including processing aspects (solvent-to-solid ratio, solvent properties, and stirring effects), microwave factors (irradiation power, extraction time, and extraction temperature), and plant material factors (plant type, particle size, moisture content, etc.) [27,28]. Therefore, all these parameters need to be optimized to ensure a high quality extraction [45].

Although this procedure is very fast compared to other extraction methods, the expensive equipment and high energy consumption are important drawbacks when used at industrial scales. Furthermore, the temperature increases in samples could cause partial degradation of some important phytochemicals [38]. Thus, thermolabile compounds such as polyphenols and other hydroxyl-type substituents could be degraded by the high temperatures achieved during MWAE, which has mainly been attributed to oxidation and degradation reactions, resulting in poor recovery rates [27,28]. This problem can be overcome by optimizing the extraction time. Thus, to limit disruption of the structural integrity and degradation of phytochemicals, the extraction time should be carefully manipulated (shortened) and also extraction cycles can be employed to reduce these degradation processes [27]. In a recent study, MWAE was used for the extraction of antioxidant compounds from *Decatropis bicolor*, which showed that this technology dramatically reduced the extraction time and improved the extract activity in comparison with a conventional extraction procedure [44]. In this study, the authors optimized the extraction conditions, such as MW power (30%), extraction time (2 min), and sample concentration (2%), which led to an extraction that was much faster than the conventional method.

As well as extracting hydrophilic compounds such as polyphenols [46], the use of MWAE has also been used to obtain essential oils. This procedure dramatically reduces the extraction time (minutes rather than hours) and solvent amounts, lowers the energy consumption and costs, and increases the essential oil yields in comparison with conventional procedures (hydrodistillation) [30]. Conversely, it increases the extraction of unwanted compounds due to severe thermal stress and high pressures [32].

The MWAE technology has an important advantage compared with other emerging techniques, since it possesses the ability to extract phytochemicals, especially oils, without the use of any solvent [26]. This technique is referred to as solvent-free microwave extraction or “dry distillation” [19,30]. In this case, plant material is placed in the microwave reactor without any solvent and then microwaved. The high temperatures and pressures produced by MWAE in the plant material water distend the plant cells and induce the rupture of the plant oil glands. Then, the released essential oil is evaporated in situ by the plant water and condensed outside of the microwave oven through a cooling system [26,30]. In this case, in addition to the time reduction, the low amounts of water present limit hydrolysis, esterification and oxidation of essential oil compounds [30].

#### 2.2.3. Pulsed Electric Field-Assisted Extraction (PEFAE)

During the last decade, PEFAE has been shown to be a useful technique for enhancing extraction efficiency. This efficient non-thermal procedure is suitable for the extraction of phytochemicals. This technique presents some important advantages over conventional methods, since it improves extraction yield, as well as decreasing processing times, energy consumption and degradation of thermolabile compounds [38,41]. In addition, it can be applied as a pre-treatment or also as an extraction assistant, thus facilitating the efficacy of other extraction technologies.

PEFAE is based on the application of intermittent high-voltage pulses (0.5–50 kV/cm), for very short intervals (micro or milliseconds) [41]. Under these conditions, the electric field disrupts the cell membranes (electroporation) [28]. Depending on the treatment intensity, the physical changes (pores) of cell membranes produced during electroporation could be transitory or permanent [41]. Moreover, the precise action of PEFAE in cytoplasmic membrane can improve the selective release of target compounds. It is also important to highlight that during treatment, the temperature remains practically unchanged, which makes PEFAE an ideal technology for the extraction of thermally-sensitive compounds [28]. As mentioned earlier, the formation of pores in the cell membranes facilitates the extraction process. This structural change increases the permeability, promotes the release of intracellular substances, and enhances the mass transfer between solutes and solvent [26,38,47]. However, the effectiveness of this technique depends on process temperature, the strength of the electric field, pulse intensity, energy input and sample characteristics [28].

The application of PEFAE has been shown to produce promising results in the extraction of both hydrophilic and lipophilic compounds from plant materials. For example, the use of PEFAE increases the extraction yields and quality (antioxidant and/or antimicrobial activity) of betalains [48], phenolic compounds [49] and essential oils [50]. For some applications, PEFAE has advantages over USAE and MWAE. PEFAE is less energy demanding (thereby reducing costs and energy consumption) and it does not increase the extraction temperature, which protects thermo-sensible compounds from degradation [38]. In addition, PEFAE can be implemented in a continuous flow process, allowing easy scale up to industry processing capacities [41]. However, as a new technique, the specific equipment is also expensive [38]. The simplicity and speed of PEFAE mean it can be combined with other extraction methods to improve their efficacy [41] Thus, the use of PEFAE as pre-treatment or in combination with other extraction procedures can improve the extraction efficiency of hydrophilic compounds (such as phenolics) and hydrophobic ones (such as essential oils). Finally, a low pulse duration with a high pulse interval may be used to improve the extraction efficiency [38].

#### 2.2.4. Supercritical Fluid-Assisted Extraction (SFAE)

SFAE has been reported to exhibit a high effectiveness for phytochemical extraction. Supercritical fluids have characteristics of both liquids and gasses under specific pressure and temperature ranges, which confer unique viscosity, density and solvation properties [27]. These simultaneous properties (liquid/gas) improved the mass transfer rate of target compounds and facilitate the extraction of phytochemicals [27,35]. Supercritical fluids can easily penetrate through plant matrices due to their low viscosity and high diffusivity like a gas and efficiently dissolve the solutes like a liquid.

Although several types of solvents can be employed in SFAE, CO_2_ is generally preferable because it is generally recognized as safe (GRAS), cheap, inert, widely available, non-toxic, and non-flammable [32,35]. Moreover, CO_2_ is easily removed from the extract by simple pressure reduction (evaporation) and could be reused after condensation. Therefore, CO_2_-SFAE is considered a safe and green technology since it did not use toxic organic solvents [35].

CO_2_-SFAE is an efficient and convenient extraction technique to obtain lipophilic compounds from plant materials, such as essential oils, oleoresins, vitamins or carotenoids [10,26,40]. This is because the non-polar character of CO_2_ makes it suitable for extracting these lipophilic compounds [10,32,33]. However, the low solubility of other important compounds (polar molecules) limits the use of pure CO_2_ as a solvent in SFAE. To overcome this limitation, the addition of co-solvents to the CO_2_ flow has been widely used. These co-solvents modify the polarity of the supercritical fluids, thus selectivity can be adapted depending on the compound to be extracted [10,51]. Several co-solvents can be used in SFAE, but ethanol (food-grade) is the most frequently used, since it meets green requirements and is considered as GRAS [27,35].

Although the use of co-solvents generally improves polyphenols extraction, several recent research studies have applied both pure CO_2_ and co-solvent-modified CO_2_ for phenolic compound extractions [52]. Moreover, SFAE was proposed as a sustainable alternative extraction technique, since it showed higher polyphenol extraction yields and extracts with higher antioxidant activity, while dramatically reducing the extraction time and solvent use in comparison with conventional techniques [15,51]. However, it is difficult to implement this technology at a large commercial scale due to the large amounts of co-solvents required. Consequently, it was recently proposed that CO_2_ should be used as the only solvent to extract polyphenols from produce using SFAE [53]. These authors optimized the temperature, pressure, and solvent flow required to isolate phytochemicals from alfalfa and showed that SFAE could be used to efficiently isolate both phenolic acids and flavonoids.

The efficiency of the extraction process is highly influenced by the extraction temperature, pressure, and time; co-solvent use (polarity); supercritical fluid flow rate; and CO_2_ density [35]. The CO_2_ density can be easily modified by adjusting the temperature and pressure of the extraction apparatus [40]. Moreover, a convenient selection of temperature and pressure also minimizes the extraction of unwanted compounds from plant matrices [27].

Particle size is another important factor that affects SFAE efficiency during phytochemical extraction. In general, a greater contact surface between the plant matrix and the solvent enhances extraction. The specific surface area of a material increases as its particle size decreases. In practice, however, the particle size has to be optimized, since if it is too low it can lead to agglomeration, thereby decreasing the surface area and reducing the extraction efficiency [27,40].

Therefore, taking into account all aforementioned aspects, SFAE reduces processing times, reduces the use of toxic solvents, increases safety, improves selectivity, and presents similar or better extraction yields [27,28,35] compared with conventional extraction methods. The absence of both oxygen and light, as well as the relatively low temperatures used during extraction, also minimize the degradation of phytochemicals [10,40]. The main drawbacks of this technology are the expensive equipment necessary and the difficulty of extracting polar phytochemicals, although this problem can be solved by using appropriate co-solvents as mentioned earlier [37].

However, it is clear that the SFAE conditions should be carefully tested and optimized from each plant material, in order to obtain the highest extraction yields and the best quality extracts [40].

#### 2.2.5. Combined Emerging Techniques

Recently, researchers have proposed using combinations of multiple emerging technologies, in simultaneous or sequential steps, which exerts synergistic effects on the extraction process. In this case, the positive effects of each technology are combined and could be effectively used in the extraction of important phytochemicals [26]. The combination of extraction techniques allows one to improve extraction yields and decrease the consumption of solvents and energy.

In a recent review, the authors conclude that simultaneous irradiation with ultrasound and microwaves is the most efficient combined technique for fast extraction [26]. In this technique, also known as UMAE, the ultrasound probe is placed into the sample, which is in a microwave oven. Thus, simultaneous treatments facilitate the diffusion of the solvent into the cells, analyte solvation and increase the solubility of the phytochemicals, which results in a faster extraction with similar or better yields. Due to the dramatically short extraction time and high efficiency, UMAE has been proposed as a potential technique for industrial purposes for the extraction of phytochemicals due to its potential advantages [37].

The combination of PEFAE and USAE has also been investigated. In this case, the pre-treatment with PEF and the subsequent extraction using USAE had synergetic effects, which enhanced the extraction yields of phenolic compounds from raspberry [54]. In a more recent study, the use of PEF as a pre-treatment step in USAE also enhanced the recovery of phenolic compounds and antioxidant capacity of rosemary and thyme by-products compared to USAE individually [55]. PEF has also been used as a pre-treatment for the extraction of phytochemicals from rosemary residue obtained after wet steam distillation [56]. The authors applied a field strength of 5.2 kV/cm in the form of 1000 pulses of 15 µs each. Then, the pre-treated samples were extracted using conventional and USAE techniques. The USAE extraction step enhanced the total phenolic yield and the antioxidant capacity of the extracts [56], which demonstrated its effectiveness.

Another promising combination is the use of ultrasound or microwave radiation as SFE-adjuvant options [40]. The use of supercritical CO_2_ and low-frequency ultrasound strongly increased the mass transfer from solid materials to fluids [57]. Moreover, the total yield increased by up to 30% with the use of ultrasound, which could be explained by the turbulence created by ultrasonic vibrations, and also by the cellular damage produced by cavitation effects. In this case, ultrasound and SFAE were applied simultaneously, but sequential steps of different emerging techniques would also be interesting. For instance, a short (30 s) microwave pre-treatment of seeds significantly increased the oil yield extracted with SFAE, while still maintaining good oil quality [58].

## 3. Characterization of Plant-Based Bioactive Agents

### 3.1. Conventional and Spectrophotometric Methodologies

Indirect measurement of phytochemicals has been carried out for decades. These indirect determinations are based on the measurement of the total antioxidant capacity, usually involving a redox reaction with the oxidant [59]. The measurement of antioxidant capacity permits determining the ability of certain molecules to eliminate free radicals or to transfer an electron to reduce an oxidant [60]. Generally, the methods for determining antioxidant properties of plant extracts can be divided according to the chemical reactions involved into hydrogen atom transfer-based, electron transfer-based and mixed mode techniques [61,62]. The most common techniques to determine the total antioxidant capacity are ferric reducing antioxidant power (FRAP) (using iron) and copper reduction (CUPRAC) (using cooper) assays, the 2,2′-azino-bis (3-ethylbenzothiazoline-6-sulphonic acid) (ABTS), Trolox equivalent antioxidant capacity, 2,2-diphenyl-1-picrylhydrazyl (DPPH), oxygen radical absorbance capacity (ORAC), total radical-trapping antioxidant parameter (TRAP), total oxyradical scavenging capacity (TOSC) and in vitro phosphomolybdenum assay. Although numerous tests are available to measure the antioxidant activity of phytochemicals, there is no single one that can reliably predict the in vivo antioxidant capacity. In fact, the antioxidant activity of phytochemicals should usually be determined using a combination of complementary antioxidant tests that are most appropriate for the target substance [63]. The use of various antioxidant methods helps to understand which type or types of mechanisms are involved in the activity of plant extracts. However, the lack of standardization of the methods makes it difficult to compare the results of different studies. Furthermore, the variations within some methods, as well as the units in which the results are expressed, are significant drawbacks of the measurement of total antioxidant capacity. In fact, these drawbacks have not been solved after 25 years of research [64]. Despite their limitations, the DPPH, ABTS, ORAC, FRAP and phosphomolybdenum assays are widely used to assess the antioxidant capacity of botanical extracts [59,65,66,67]. Thus, the total antioxidant capacity (using the aforementioned methods) is normally assayed before the use of the plant extracts and phytochemicals, in order to know their functionally and technological aptitudes for food application [17,22].

Groups of phytochemicals can also be characterized directly using spectrophotometric techniques. For instance, the determination of the total content of phenolic compounds (TPC) [68], betalains [69,70], carotenoids [71], anthocyanins and chlorophylls [22] has been widely used for the characterization of plant extracts, although they have the disadvantage that it only gives general information, and it is not possible to determine the specific constituents of the extract. On the other hand, some compounds, such as betacyanins, betaxanthins, or some carotenoids such as β-carotene or lycopene can be determined using spectrophotometric techniques. However, there may be interferences in the determination since many other compounds can absorb in the same wavelength range, so the amount of these phytochemicals could be overestimated.

In the case of essential oils, some classical analytical techniques have been used to verify oil purity, including density and refractive index measurements, the determination of polar substances, and the measurement of melting and congealing points. Additionally, the aldehydes (bisulfide method) and ketones (neutral sulfide test) content or the solubility test in ethanol are also frequent analysis in essential oils characterization [32].

### 3.2. Chromatographic and Mass Spectrometric Methodologies

Although conventional methodologies are very useful for screening purposes and giving us a general idea of the properties of extracts obtained from plants, the use of chromatographic and mass spectrometric techniques is more appropriate for the more precise identification and quantification of bioactive molecules. This is because extracts are normally molecular complex mixtures containing several phytochemicals, thus, the use of selective, sensitive, and versatile analytical techniques is necessary [72].

Several methods using liquid chromatography with diode array detector (LC–DAD) have been used for the determination of phytochemicals, operating in reverse phase for hydrophilic compounds (for example, for the determination of betalains or polyphenols) or in normal phase for lipophilic compounds (for example, carotenoids or tocopherols). In this case, several advantages were observed in comparison with spectrophotometric methods, since chromatographic methodologies ensure the correct separation/resolution of specific compounds, and therefore, their correct identification and quantification. Moreover, the specific compounds/isomers can also be quantified. For example, the use of LC–DAD allows the quantification of different red betalains (amaranthine, isoamaranthine, probetanin, betanin, isobetanin, betanidin, and isobetanidin) that compose the betacyanins fraction of different plants, while the use of spectrophotometric techniques only allows the quantification of “total” betacyanins [69,70]. LC–DAD is one of the most used analytical techniques for the determination of polyphenols and polyphenol-rich extracts [45,73,74].

The use of liquid chromatography combined with mass spectrometry is the most convenient and powerful technique to characterize complex botanical extracts. For instance, LC–TOF/MS and LC–ESI–MS were able to identify more than 32 isomers in betalains [69,75], while DAD could not discriminate them all. Although LC–DAD is a relatively simple technique to determine polyphenols in botanical extracts, it mainly discriminates and identifies molecules based on their retention time (using reference standards) and UV spectra [72]. Thus, LC–MS and LC–MS/MS techniques have been increasing in order to more thoroughly characterize polyphenol-rich extracts. Several combinations were used in a comprehensive polyphenol identification and quantification, including liquid chromatography hybrid linear ion trap quadrupole-Orbitrap-mass spectrometry (LC–LTQ–Orbitrap–MS) [76] or LC–QTOF/MS [75]. Furthermore, in some cases, the use of a single quadrupole mass spectrometer is not selective enough, thus tandem mass spectrometry (MS/MS) is needed to achieve noise reduction and improve sensitivity by exploiting a multiple reaction monitoring (MRM) scan mode [72]. Several researchers have successfully used this technique [55,65,77]. Thus, LC–MS/MS is currently the most powerful technique for the correct identification and quantification of phytochemicals.

On the other hand, although both gas and liquid chromatography could be used for essential oil analysis, the gas chromatography technique is preferably due to the volatile nature of their constituents [33]. Gas chromatography (GC) using non-polar fused silica capillary columns are suitable for chromatographic separation and resolution of these materials. Multiple kinds of detectors can be utilized to quantify the separated peaks, including flame ionization detector (FID) and mass spectrometer (MS). Among these detectors, the FID needs standards for the correct identification of the compounds (based on the retention time), while the use of mass spectrometry does not need any standards since the use of mass spectrum comparison with international spectrum libraries ensures correct identification of all essential oil constituents. This fact is a major advantage of MS compared to FID, since it even allows the identification of different isomers of the same compound, and using different tools (for example, deconvolution) allows separating compounds that coelute. Thus, gas chromatography coupled with mass a spectrometer (GC–MS) is the most common method currently used in the determination of essential oil composition. Several authors have used this powerful technique to determine the composition of complex mixtures of phenylpropanoid derivatives and terpenoids [78,79,80,81], which are the main constituents of essential oils [31,33] and responsible for their characteristic odor [30]. Furthermore, solid-phase microextraction (SPME) is a solvent-free isolation technique that allows the extraction and concentration of volatile compounds from vial headspaces. Therefore, the combination of SPME and GC–MS is a reliable and fast method to determine essential oil composition, since it improves the detection limits and resolution [30].

In general, methods involving chromatography coupled to mass spectrometry are currently the best options for the complete characterization of botanical extracts, but they are relatively expensive, which limits their utilization in some laboratories.

## 4. Encapsulation of Plant-Based Bioactive Agents

Many bioactive agents isolated from botanical sources cannot be used directly because of their poor water solubility, low chemical stability, or limited biological activity. These characteristics can often be improved by encapsulating the bioactive agents into colloidal particles. These particles can be designed to increase the water dispersibility, shelf-life, and activity of bioactive agents, provided they are designed correctly. In this section, an overview of encapsulation technologies that can be used for this purpose is given.

### 4.1. Particle Design

It is important to carefully design the colloidal particles used to encapsulate bioactive agents for specific applications. The design of particle properties depends on numerous factors, which are summarized here [82,83,84,85]:

- *Bioactive component:* The nature of the bioactive component to be encapsulated, such as its molecular weight, polarity, charge, solubility, physical state, and chemical stability, will impact that most appropriate type of colloidal particle. For instance, a hydrophobic bioactive will typically be encapsulated within a colloidal particle that has some hydrophobic domains inside, such as the core of a micelle, lipid droplet, or solid lipid nanoparticle or the bilayers of liposomes. Conversely, a hydrophilic bioactive will typically need to be encapsulated within a colloidal particle that has some hydrophilic domains, such as the aqueous interior of liposomes or biopolymer microgels. The electrical charge of bioactives may also be important as this may determine their ability to be retained/released from colloidal particles. For instance, a negatively charged bioactive may stick to a protein-based particle below the isoelectric point where the proteins are cationic and can therefore be retained, but not above the isoelectric point where the proteins are anionic and can therefore be released. If a bioactive is highly sensitive to chemical degradation (such as oxidation), it may be important to select colloidal particles that have good antioxidant properties.
- *End product matrix*: The nature of the end product that the bioactive component is going to be incorporated into also impacts the most suitable delivery system for a particular application, e.g., food, beverage, capsule, pill, or packaging material. These end products vary in their physicochemical and sensory properties, such as their optical properties (e.g., color and opacity), rheology (e.g., fluid, gel, or solid), shelf-life (e.g., days to years), flavor profile (smell and taste). The colloidal delivery system must therefore be compatible with the properties of the end product. For instance, a delivery system that is optically transparent (such as a nanoemulsion, microemulsion, or biopolymer nanoparticles with particle sizes below 50 nm) may be required in foods or beverages that are transparent (such as soft drinks or fortified waters). Small particles may also be needed in end products that have low viscosities to avoid creaming or sedimentation during storage. The size of the particles may also have to be controlled to alter the mouthfeel of the product. For instance, particles larger than approximately 50 microns can be sensed as discrete objects by the tongue.
- *Environmental conditions:* The type of environmental conditions, such as pH, ionic strength, temperature, light, oxygen, ingredient interactions, and mechanical forces, that the fortified end product is exposed to during its manufacture, storage, transport, and utilization must also be taken into account. It may be necessary to design the colloidal delivery system so that it is resistant to any environmental stressors that might promote physical or chemical destabilization. As an example, most protein-based colloidal particles tend to aggregate near their isoelectric point or at high ionic strengths and so they may not be used in food or beverage products that have pH values or salt contents in these ranges.

In addition, it is important to select ingredients that are legally accepted in the intended country of use, that are economically viable, and that are label friendly. This may greatly limit the types of ingredients that can be used to formulate a delivery system.

### 4.2. Particle-Building Ingredients

A wide range of different botanical ingredients can be used to assemble plant-based colloidal delivery systems for bioactive agents. A brief summary of some of the most important ones is given here [86]:

- *Proteins*: In general, proteins are extremely versatile ingredients for constructing colloidal particles because of their diverse range of functional attributes, including emulsification, foaming, gelation, film forming, and structure forming. As a result, they have been used to assemble many different kinds of colloidal delivery systems, including nanoemulsions, protein nanoparticles, and microgels. Proteins consist of polypeptide chains that vary in the number, type, and sequence of amino acids present. In some cases, proteins may also be physically or covalently attached to other constituents that alter their functional properties, such as carbohydrates (glycoproteins), lipids (lipoproteins), heme groups (heme proteins), or metals (metalloproteins). The functionality of proteins is strongly linked to their molecular conformations and aggregation states. Most commonly used plant proteins have globular structures, which may be denatured during their isolation and purification, thereby altering their functional performance. Moreover, many plant proteins are physically or covalently bound to other proteins in nature. The aggregation state of these proteins may be changed during isolation and purification, which can also impact their functionality.
- *Polysaccharides*: Polysaccharides are also versatile food ingredients that can be used to construct a variety of colloidal delivery systems due to their functional attributes, such as thickening, gelling, emulsifying, and structure forming. In particular, they are commonly used as scaffolds in microgels and emulsifiers in nanoemulsions and emulsions. Polysaccharides consist of chains of monosaccharides covalently linked together by different kinds of glycosidic bonds. The number, type, bonding, and sequence of the monosaccharides largely determine the functional attributes of a polysaccharide. Polysaccharides vary in their molecular weights, branching, polarities, and electrical charges, which impacts their ability to form colloidal particles. Electrostatic interactions are often used to create structures. For instance, microgels may be formed by mixing a positive protein with a negative polysaccharide or by mixing a negative polysaccharide with positive multivalent ions to create biopolymer networks that trap water. In general, polysaccharides can form gels through a variety of mechanisms, which is useful for different applications: heating, cooling, salt addition, pH changes, and enzymes. Polysaccharides also vary in their digestion and fermentation properties in the human gut. Some polysaccharides may be digested within the upper gastrointestinal tract, such as starch in the mouth, stomach, and small intestine. Conversely, other polysaccharides (dietary fibers) are only digested and fermented when they reach the colon. Some of these dietary fibers may actually pass through the full gastrointestinal tract (GIT) with little or no digestion and fermentation. Knowledge of the gastrointestinal behavior of polysaccharides may be important for developing colloidal delivery systems for the oral delivery of nutrients, nutraceuticals, or probiotics to specific regions of the GIT.
- *Lipids*: Lipids are substances that are insoluble in water but soluble in organic solvents. A wide variety of lipids can be isolated from botanical sources, including acylglycerols, phospholipids, sterols, and waxes. The most commonly used plant-based lipids are triacylglycerols, which consists of three fatty acids covalently attached to a glycerol molecule. The chain length, unsaturation, and position of the fatty acids differ depending on the source of the lipids. As a result, different edible triacylglycerols have different physicochemical properties and nutritional attributes, such as viscosity, solidity, oxidation stability, digestibility, and health effects. Lipids are commonly utilized in the formation of nanoemulsions, emulsions, and solid lipid nanoparticles that are used to encapsulate bioactives. Phospholipids, which consist of two fatty acids and a phosphate group attached to a glycerol molecule, are commonly used to form nanoemulsions, emulsions, and liposomes. They tend to self-assemble into bilayer structures that have a hydrophobic interior and hydrophilic exterior. Hydrophobic bioactives can be solubilized within the non-polar regions, whereas hydrophilic bioactives can be solubilized within the polar regions. They can also be used to coat the oil droplets in emulsions and nanoemulsions because the hydrophilic head points towards water, whereas the hydrophobic tail points towards oil.

### 4.3. Encapsulation Technologies

**Figure 2:**
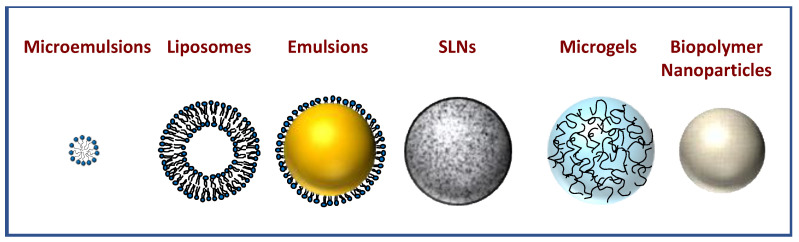
Examples of different kinds of colloidal delivery systems that can be used in foods and other end products.

A diverse range of encapsulation technologies have been developed to encapsulate bioactive agents, which vary in the ingredients and processing methods used to assemble them [82,86,87]. In this section, we review a number of the most important ones that can be used to encapsulate botanical bioactive substances (Figure 2).

#### 4.3.1. Plant-Based Micelles and Microemulsions

In general, micelles and microemulsions are colloidal particles comprised of surfactant molecules assembled into spheroid structures with the non-polar tails facing inwards (away from water) and the polar heads facing outwards (toward water) (Figure 2) [88,89,90,91]. Typically, micelles are only formulated using surfactants, whereas microemulsions may also contain co-surfactants and oils. Micelles typically have diameters of approximately 5 to 20 nm, whereas microemulsions have diameters of approximately 20 to 100 nm. Hydrophobic bioactives are typically solubilized within the non-polar domains within the interior of these colloidal particles, i.e., between the surfactant tails or within a central lipid core. Amphiphilic bioactives may also be solubilized between the surfactant tails. Micelles and microemulsions are thermodynamically stable because they have a lower free energy than the separated components (oil, water, and surfactant). It should be noted, however, that they are only thermodynamically stable over a certain compositional and environmental (pH, ionic strength, and temperature) range, and tend to breakdown if they move out of this range. In principle, micelles and microemulsions should form spontaneously when the different components are brought into contact because of the negative free energy associated with their assembly. In practice, it is often necessary to apply some form of external energy (such as mixing or shearing) to overcome activation energies associated with self-assembly of the surfactants in water [92].

Traditionally, micelles and microemulsions are formed from small molecule synthetic surfactants, such as Tweens and Spans. Nevertheless, they may also be formed from some plant-derived surfactants, such as the saponins derived from quillaja or tea trees. These surfactants have a hydrophilic part and a hydrophobic part, which allows them to self-assemble into micelles or microemulsions in aqueous solutions.

#### 4.3.2. Plant-Based Nanoliposomes and Liposomes

In general, liposomal systems consist of colloidal particles that are made up of one or more concentric phospholipid bilayers (Figure 2) [93,94,95,96,97,98]. Nanoliposomes (*d* < 100 nm) can be distinguished from liposomes (*d* > 100 nm) due to their smaller diameters. Nevertheless, both types of systems are only metastable, i.e., they tend to breakdown over time because the separated state has a lower free energy than the liposomal system. Even so, the formation of the phospholipid bilayers does occur spontaneously because of the hydrophobic effect. Liposomal systems can be used to encapsulate amphiphilic, hydrophilic, or lipophilic bioactive substances because they have regions with different polarities. Hydrophilic bioactives can be incorporated into the aqueous interior of liposomal systems or between the polar head groups of the phospholipids, whereas lipophilic and amphiphilic bioactives can be incorporated within the hydrophobic domains formed by the phospholipid tails (Figure 2). Liposomal systems can be further characterized by their tendency to form single (unilamellar) or multiple (multilamellar) phospholipid bilayers. The formation of these different structures is governed by the ingredient formulation and processing methods utilized in their assembly.

Liposomal systems can be formulated entirely from plant-derived ingredients, such as soybean or sunflower lecithin, which means that they are suitable for application in plant-based foods and other functional materials. This kind of colloidal dispersion can be fabricated using a variety of approaches, which vary in their efficacy and suitable for large-scale production. Some of the most commonly employed methods for producing liposomal systems are solvent evaporation/rehydration, solvent injection, and microfluidization methods [86]. The main disadvantages of liposomal systems are that it is often challenging to incorporate high amounts of bioactive components, the encapsulation efficiency is relatively low, and they have a tendency to breakdown over time, particularly when exposed to extreme conditions, such as high salt concentrations, acidic conditions, and elevated temperatures.

#### 4.3.3. Plant-Based Nanoemulsions and Emulsions

Nanoemulsions and emulsions both consist of small droplets of one fluid (the “dispersed phase”) distributed throughout another immiscible fluid (the “continuous phase”) [94] (Figure 2). In the food industry, these two fluids are usually oil and water. An oil-in-water (O/W) system is formed when the oil phase forms the droplets, whereas a water-in-oil (W/O) system is formed when the water phases forms the droplets. Typically, the droplets are coated by a layer of emulsifier molecules to prevent them from aggregating with each other. The free energy of nanoemulsions and emulsions is higher than that of the separated phases, and so they are thermodynamically unfavorable [99,100]. These systems must therefore be designed to ensure that they are metastable, i.e., have a sufficiently long shelf-life for the intended application. This usually involves controlling the droplet composition, concentration, and size, as well as by using suitable additives such as emulsifiers, thickeners, gelling agents, weighting agents, and ripening inhibitors [99,100]. Conventionally, the mean diameter of the droplets in nanoemulsions is below 100 nm, whereas it is above this value for emulsions. The smaller dimensions of the droplets within nanoemulsions have some important consequences for their functional attributes, typically leading to greater optical transparency, improved resistance to aggregation and gravitational separation, and a higher bioavailability of any substances encapsulated within them.

Nanoemulsions and emulsions can be created using a broad spectrum of methods, which can be classified as high-energy or low-energy approaches [86]. High-energy methods employ specially designed mechanical devices, such as high-shear mixers, colloid mills, microfluidizers, sonicators, or high-pressure valve homogenizers, to generate intense disruptive forces that break up the oil and water phases. In contrast, low-energy methods rely on the spontaneous formation of small droplets when certain oil, water, and surfactant mixtures are treated in a specific manner, e.g., their composition or temperature is changed. These latter methods include phase inversion temperature, spontaneous emulsification, and emulsion inversion point methods. Emulsions and nanoemulsions can be formulated entirely from plant-based ingredients, such as plant-derived oils (such as corn, sunflower, and flaxseed oil), emulsifiers (such as soy protein, sunflower lecithin, or modified starch), and stabilizers (such as pectin or cellulose).

#### 4.3.4. Plant-Based Solid Lipid Nanoparticles and Nanostructured Lipid Carriers

Solid lipid nanoparticles (SLNs) and nanostructured lipid carriers (NLCs) have structures that are fairly similar to those found in O/W nanoemulsions, but the oil droplets are fully or partially crystallized, respectively (Figure 2) [101,102]. Indeed, these systems are often formed by creating an O/W nanoemulsion at a temperature above the melting point of the oil phase and then cooling it to crystallize the droplets. Like nanoemulsions, the lipid particles in SLNs and NLCs are stabilized by coating them with a suitable emulsifier. One of the advantages of SLNs and NLCs over nanoemulsions is that crystallizing the oil phase retards the diffusion of molecules inside the lipid particles, which can improve the retention of encapsulated substances, as well as protecting them from chemical degradation. It should be noted, however, that the oil phase must be carefully designed to achieve this. If the oil phase forms a crystalline structure that is too regular, then any encapsulated bioactive agents may be expelled because they cannot be accommodated within the crystals. In addition, the formation of a highly regular crystalline phase can cause lipid particles to undergo a transition from a spherical to an irregular shape, thereby increasing their oil–water surface area. Consequently, there may not be enough emulsifier molecules present to saturate the lipid particle surfaces, thereby promoting particle aggregation [103]. SLNs are particularly prone to these problems because they contain fully crystalline nanoparticles. Conversely, NLCs are less prone because the lipid phase is selected to be only partially crystalline after it solidifies, thereby preventing the expulsion of encapsulated bioactive substances and inhibiting particle morphology changes. NLCs and SLNs can be fabricated entirely from plant-based lipids (such as coconut or palm oil), emulsifiers (such as plant proteins, polysaccharides, and phospholipids), and stabilizers (such as plant proteins and polysaccharides).

#### 4.3.5. Plant-Based Biopolymer Nanoparticles and Microgels

Plant-based proteins and polysaccharides can be used to assemble biopolymer nanoparticles and microgels (Figure 2) [104,105,106]. Biopolymer nanoparticles mainly consist of tightly packed proteins and/or polysaccharide molecules, with only a little water. Conversely, biopolymer microgels consist of a protein and/or biopolymer network that traps large amounts of water. The biopolymer molecules in these particles are typically held together by physical or chemical bonds, including hydrogen bonding, hydrophobic attraction, electrostatic interactions, and disulfide bonds. The dimensions of these particles ranges from approximately 100 to 1000 nm depending on the ingredients and fabrication method used. There are a wide range of different fabrication methods available to produce biopolymer nanoparticles and microgels, including antisolvent precipitation, injection-gelation, phase separation-gelation, emulsion templating, and molding methods [104]. The composition, porosity, shape, and dimensions of biopolymer nanoparticles and microgels can be manipulated to provide desirable loading, retention, and release properties. These kinds of biopolymer nanoparticles and microgels can be fabricated from plant-based ingredients, such as plant proteins (e.g., zein, gliadin, and soy protein) and polysaccharides (e.g., alginate, carrageenan and pectin).

#### 4.3.6. Plant-Based Cyclodextrins

Cyclodextrins are another commonly used type of delivery system for active ingredients. These carbohydrate-based systems consist of rings of glucose molecules (typically 5 to 8) held together by α 1–4, glycosidic bonds. They are usually produced from starch molecules using enzymatic methods. The interior of the cyclodextrin ring is hydrophobic, which means that non-polar active molecules with appropriate molecular dimensions can be incorporated inside [107]. The size of the hydrophobic interior increases as the number of glucose units in the cyclodextrin molecule increases, which means that different bioactive agents can be accommodated. Cyclodextrins can be used to increase the water dispersibility, solubility and stability of active agents, and they are widely used in many food industrial products [107,108,109].

## 5. Application of Encapsulated Plant-Based Active Ingredients in the Meat Industry

There is great interest in using botanically-derived preservatives in the meat industry. Oxidative reactions and microbial growth are the two main degradation processes involved in the loss of quality in meat and meat products [11]. In addition to the nutritional quality loss (degradation of unsaturated fatty acids and vitamins), accumulation of toxic compounds derived from oxidation reactions and the reduction in sensory quality and consumer acceptance (rancid flavor and odor) [11], the changes in meat color are also important. The loss of the characteristic bright cherry-red color in meat and meat products is a consequence of myoglobin oxidation, which is directly related to the redox state of iron in the heme fraction of myoglobin molecule [110]. Similarly, microbial spoilage occurs in meat and meat products, which could promote the growth of pathogenic microorganisms and produce unpleasant odors, abnormal discoloration, and the presence of slime that limit the shelf-life of these products [110].

To inhibit these negative alterations in meat quality during storage, several additives are normally used by the meat industry. However, most of them are synthetic additives, which could exert negative effects on human health [19]. Moreover, the growing interest of the consumer in minimally processed food results in growing interest within the meat industry on replacing synthetic additives with natural antimicrobials and antioxidant agents [21,28]. Thus, researchers in academia and industry are carrying out studies to find new alternatives, including natural extracts (polyphenol-rich extracts, oleoresins, purified compounds, etc.) or essential oils from plant materials, which are added to the meat formulation [8,9,10,14,19,65,77] or to the packaging materials [6,110,111] to increase meat and meat products shelf-life [28]. In addition to the antioxidant and antimicrobial properties of these compounds, the use of certain extracts or phytochemicals, with a red color (anthocyanins, betalains, lycopene, etc.) could also be important to maintain sensory properties, since they also act as natural colorants [10], which increase the stability of the characteristic red color of meat and meat products. Essential oils can be also applied as natural flavors to meat products [32]. Moreover, a number of studies have also shown that pollen and pollen extracts as well as propolis can be utilized as effective preservatives in meat products [112]. In this regard, bee pollen was applied as a natural antioxidant to prevent the degradation of refrigerated sausages [113] and meatballs [114,115], while propolis extract was added to increase the shelf-life of beef and pork patties [116,117].

On the other hand, as mentioned in Section 2, phytochemicals (phenolic compounds, betalains, carotenoids, terpenoids, etc.) are the major constituents of plant materials that contribute to their antioxidant and/or antimicrobial activity. Thus, several plant materials, including roots [9], berries/fruits [14], leaves [65], seeds [7,77] or also agro-food by-products [10,15] are potential sources of these important bioactive compounds that could be used as natural and promising additives in the meat industry. These bioactive compounds may be incorporated in meats as water-soluble extracts, water-insoluble extracts (oleoresins, essential oils, etc.) and powders [14]. Thus, phytochemical-based preservatives are gaining popularity in the meat industry since they are perceived by consumers as safe and are Generally Recognized as Safe [21]. In recent decades, several researchers have therefore studied the antimicrobial and antioxidant activity of plant extracts and essential oils in various meat products [7,8,19,20,21,110]. However, it is also important to highlight that these natural extracts should not negatively influence the sensory properties of meat products, and they should be active at low concentrations, inexpensive, and stable during the manufacturing process for industrial applications. Furthermore, prior to the incorporation of the phytochemicals in meat products, evaluating the toxicity of these compounds to human cells through in vivo studies and clinical trials to better understand their potential effects on consumer health should be carried out [28].

Some recent reviews make an in-depth analysis of the direct application of different extracts [7,9,10,14,16,21] and essential oils [8,19,20] in meat products, focusing on their antioxidant and/or antimicrobial function. However, most of these reviews have not considered the application of encapsulated phytochemicals within the meat industry. In this section, recent studies on the effects of encapsulated phytochemicals on the main degradative phenomena of meat and meat products are therefore reviewed.

Encapsulation of bioactive compounds usually increases their stability during storage and processing by increasing their resistance to environmental conditions, such as pH changes, high temperatures, light exposure, and oxidative conditions. They are also being explored for their ability to control the release of phytochemicals within meat products. These studies have shown that encapsulation technologies offer a promising strategy for improving the quality of meat products, increasing their nutritional properties, and limiting degradation processes (such as microbial contamination and oxidative reactions) [12,13]. Table 2 summarized some recent studies where encapsulated phytochemicals (either as extract or as essential oil) were incorporated into meat or meat products formulations to improve their quality, shelf-life or safety.

The incorporation of nano-encapsulated rosemary extract into fresh beef was shown to increase its shelf-life [118]. In this study, the immersion of the beef into solutions containing either 800 or 1600 ppm of non-encapsulated or encapsulated rosemary extract on lipid oxidation and microbial growth was examined. The primary (peroxide value) and secondary (TBARs) lipid oxidation-derived compounds were significantly reduced in the treated samples compared to the control. Moreover, the encapsulated extract had a higher antioxidant activity than the non-encapsulated extract [118]. The rosemary extract also had a powerful antimicrobial effect on the meat during storage. The control samples presented the highest total viable counts, while the samples treated with 1600 ppm nanoencapsulated extract had the lowest values. Additionally, during storage, the control samples showed pronounced changes in the color parameters, while there were only small changes in color in the treated samples. As expected, the antioxidant and antimicrobial properties of the rosemary extract were dose-dependent. Overall, this study highlighted the ability of encapsulation to increase the antioxidant and antimicrobial capacities of natural extracts for meat applications [118].

Other authors have used orange essential oil as the continuous phase and cactus acid fruit extract as the dispersed phase of nanoemulsions [119]. This nanoemulsion formulation was employed in the manufacture of emulsified meat systems at different levels (between 0 and 5%). After manufacture, these authors studied the total phenolic content and the antioxidant activity (DPPH and ABTS) of the meat systems. As expected, they found that these values increased in proportion to the amount of nano-encapsulated extract incorporated into the meat. This fact was in accordance with the lipid oxidation results, which showed that the incorporation of nanoemulsions (with antioxidants from cactus acid fruit and orange essential oils) reduced the secondary lipid oxidation products (TBARs) compared to control samples.

Color parameters are one of the most important quality attributes of meat products that determine overall consumer acceptance. Cooked ham is a widely consumed meat product that is expected to have a characteristic pinky color. Some researchers have proposed the use of natural dyes as alternative colorants to synthetic ones in cooked ham [120]. In this research, the use of encapsulated (with maltodextrin) and non-encapsulated anthocyanin-rich extracts (obtained from hibiscus and red radish) and betalain-rich extracts (obtained from red beetroot) were tested and compared to the results with commercial carmine dye (E120). Ham products containing encapsulated hibiscus extract had the most similar color (instrumental color and visual aspect) to that obtained with the commercial dye. In contrast, cooked ham formulated with non-encapsulated red beetroot extract had the best color, in comparison with encapsulated beetroot and the other extracts. The authors conclude, the use of non-encapsulated red beetroot extract provides the intended color, although the use of encapsulated hibiscus extract could also be used as a natural colorant in the cooked ham product [120].

Another important and controversial synthetic additive that is widely used in the meat industry is nitrite. Great efforts are being made to limit its use, but to date, a substitute has not yet been found that has the properties and effects that nitrites exert in meat products. Therefore, the solution is the combined use of nitrites with other compounds that make it possible to reduce the nitrite content added to meat products. A recent study proposed the use of nanoencapsulated lupulon and xanthohumol as nitrite replacers in cooked beef sausage [121]. These phytochemicals were added as nanoliposomes, which released the lupulon and xanthohumol in a progressive way during storage. In this study, the authors combined different amounts of nanoliposomes and nitrite, and they found that partial nitrite replacement did not affect the proximate composition nor pH of beef sausages during storage. On the other hand, strong antimicrobial activity was observed when nanoliposomes were combined with nitrite. Additionally, in some cases (i.e., coliforms), the use of nanoliposomes alone, although presenting a high antimicrobial effect, showed less effectiveness than the combination of both preservatives. The authors related this activity with the synergistic effect of nanoliposomes and nitrite, which effectively inhibit the growth of different microbial species such as *C. perfringers* and coliforms, and reduce the total count and mold and yeast count [121]. Additionally, the use of nanoliposomes + nitrite was also shown to prevent lipid oxidation in cooked beef sausages. In this case, the samples formulated with 30 ppm nitrite + 150 ppm nanoliposomes or 60 ppm nitrite + 100 ppm nanoliposomes presented the lowest oxidative degradation. Similarly, the use of only 30 ppm nitrite in combination with nanoliposomes also was sufficient for inducing proper redness, demonstrating that this synthetic preservative could be effectively replaced by natural phytochemicals encapsulated in nanoliposomes. In fact, in a sensory evaluation, the control samples (with 120 ppm of nitrite) and the different combination of nanoliposomes and nitrite (between 30 and 90 ppm) did not show differences in taste, odor, texture, color and consumer acceptance, which confirms the potential use of this strategy for partial replacement of nitrites. Thus, the use of nitrite was observed to be critical, but a significant reduction could be feasible to limit the use of this preservative without affecting the quality of meat products [121].

The use of encapsulated essential oils also showed promising antimicrobial abilities. Microencapsulated *Allium sativum* essential oil (maltodextrin/Gum arabic) in minced meat [122] and thyme essential oil (casein/maltodextrin) in hamburger-like meat [123] presented a strong antimicrobial activity. *A. sativum* microcapsules (containing 20% of essential oil) added at 0.1% to minced meat inhibited the growth of total aerobic mesophilic flora, sulfite-reducing anaerobes, and *E. coli* [122]. Moreover, this study observed that the inhibitory effect of microcapsules increased in a dose-dependent manner between microcapsules containing from 5 to 20% of essential oil [122]. Microencapsulated thyme essential oil has been shown to inhibit the growth of thermotolerant coliforms and *E. coli* in hamburger-like meat, which confirm the effectiveness of essential oils against microbial spoilage [123]. These authors found that these microcapsules could control these microorganisms over periods of up to 14 days, and highlighted that encapsulated essential oil is more effective than its direct addition due to the slow release of volatile phytochemicals during storage [123]. Thus, essential oils microcapsules could be a good natural alternative to synthetic food additives, particularly as effective antimicrobial agents.

The application of encapsulated and non-encapsulated prickly pear extract to beef burger patties was shown to improve their quality and stability [124]. Samples treated with both non-encapsulated and encapsulated prickly pear fruit extract showed lower values of mesophilic bacteria, *Enterobacteriaceae* and *Pseudomonas* spp. in comparison to control samples. This fact demonstrated the antimicrobial activity of the prickly pear fruit extract. Additionally, the pH of the burger also was affected by the extract addition, since in treated samples the variation in pH was minimal, while in the control samples, an increase was observed during storage, probably due to the release of basic substances during microbial growth and protein degradation [124]. Likewise, the color parameters were also more stable in samples formulated with encapsulated prickly pear fruit extract, with a more intense redness in the burgers being maintained throughout storage. Therefore, these authors concluded that the addition of encapsulated extract led to more desirable color and textural features, which was attributed to their ability to inhibit microbial growth and chemical reactions [124].

The use of encapsulated leaf extracts as preservatives has also shown promising results. In this regard, olive leaf extract encapsulated in a double-emulsion system [125] and the *Laurus nobilis* leaf extract encapsulated in nanoliposomes [126] were shown to be effective natural preservatives to maintain the quality of meat systems and minced beef, respectively. In the case of the encapsulated olive leaf extract, a strong antioxidant activity was observed. In meat systems with this encapsulated extract, and under accelerated oxidative conditions, the primary and secondary lipid oxidation was inhibited. The authors attributed this effect to both antioxidant properties of the extract and also the structure of double emulsions, which hinder the oxidative reactions [125]. The concentration of oleuropein (the main phytochemical of olive leaves) in the meat systems was directly related with the antioxidant activity (measured by DPPH and FRAP) and inversely related with lipid oxidation (peroxide and TBARs values). The authors reported that the encapsulation of olive leaf extract had a positive effect on the retention of oleuropein, controlling its release and increasing its oxidative stability in comparison with non-encapsulated extract. Incorporating encapsulated extracts into meat systems has also been reported to lead to higher binding and improved texture properties [125]. Likewise, the addition of encapsulated *Laurus nobilis* leaf extract via nanoliposomes into minced beef was shown to protect it from degradative processes [126]. Non-encapsulated and encapsulated extract both exhibited a strong antioxidant activity in the minced meat, i.e., reduction in peroxide and TBARs values. Additionally, this extract also protected the meat from other important degradative reactions such as lipolysis (expressed as free fatty acids content) and proteolysis (measured by total volatile basic nitrogen). It is well known that lipolytic and proteolytic reactions are directly related to the activity of microbial enzymes. Thus, the reduction in these phenomena was related to the antimicrobial activity of this extract. The addition of the encapsulated and non-encapsulated extract were also shown to exhibit powerful inhibition of *E. coli* and *S. aureus* growth and to reduce the total viable and psychrotrophic bacteria count [126]. Encapsulation was reported to enhance the protective properties of the bay leaf extract. According to the results of this study, the use of 1000 ppm of encapsulated extract presented similar antioxidant and antimicrobial activity as 1500 ppm non-encapsulated extract. Therefore, encapsulation appears to enhance the preservative activity of the extracts and prolong their effectiveness during storage [126]. However, the sensory evaluation indicated that the addition of bay leaf extract to minced meat resulted in a decrease in the taste and overall acceptance scores, while color and odor scores were the same for treated and control samples. Despite these differences, the authors concluded that all treatments had sensory ratings approved by the evaluators [126].

Free and encapsulated (nanoliposomes) bioactive peptides obtained from quinoa have been used to increase the shelf-life and quality of burgers [127]. In this study, the incorporation of bioactive peptides showed a protective effect against lipid oxidation (peroxide and TBARs values) and proteolytic reactions (total volatile basic nitrogen). In addition, microbial assays showed that the quinoa bioactive peptides reduced the total bacterial count and growth of *S. aureus*, mold, and yeast [127], which could explain the lowest values of the proteolytic phenomenon in treated burgers. Thus, encapsulation also enhanced the efficacy of bioactive peptides by increasing their antioxidant and antimicrobial properties. Indeed, burgers that contained nanoliposome-encapsulated peptides exhibit higher preservative effects than those formulated with the same amount of free peptides. Thus, these authors concluded that the use of peptides obtained by quinoa protein hydrolysis, and applied as nanoliposomes could be introduced as a natural preservative in the meat industry [127].

It is well known that the main antioxidant and antimicrobial properties of botanical extracts/essential oils are mostly related to the high activity of the phytochemicals present within them (including phenolic compounds, terpenoids, carotenoids, and betalains). However, recent studies suggest that encapsulation can be used to increase the efficacy of these botanical extracts in meat products, thereby extending their shelf-life, improving their quality, and enhancing their safety. Encapsulation may also have some other advantages, including the protection of phytochemicals during the processing of meat products, the masking of undesirable flavors of certain extracts/essential oils, and the controlled or sustained release of these bioactive compounds.

## 6. Conclusions and Future Perspectives

There has been growing interest among consumers in having more ethical, environmentally-friendly and sustainable products. This has led the food and other industries to explore replacing synthetic materials used in their products with natural plant-based ones. In this article, we have reviewed some of the most important botanical bioactive agents that are being explored as natural preservatives, pigments, flavors, and nutraceuticals in foods and other products. In particular, we have highlighted the importance of developing effective methods of identifying, isolating, and purifying botanical components from sustainable botanical sources. Moreover, we have provided an overview of different kinds of colloidal delivery systems that can be used to encapsulate these components with an aim of improving their water dispersibility, storage stability, and efficacy, as well as to provide sustained release profiles. Colloidal particles increase the water dispersibility of hydrophobic non-polar phytochemicals by trapping them within structures that have a hydrophobic core and a hydrophilic shell that is compatible with aqueous solutions. They enhance the chemical stability of phytochemicals by protecting them from any reactive molecules in the surrounding medium, such as acids, bases, or enzymes, or by shielding them from exposure to light or oxygen. They can be used to increase the bioavailability of hydrophobic phytochemicals by forming mixed micelles in the small intestine that can solubilize and transport them. Finally, we demonstrated the potential of encapsulated botanicals as additives using meat and meat products as an example.

Further research is required to identify new sources of botanical extracts that have improved efficacy, and which do not adversely affect food quality. Moreover, it will be important to develop extraction methods and encapsulation technologies that are commercially viable, otherwise their potential benefits will not be realized in practice. In particular, it will be important to develop cost-effective methods and technologies that can be utilized at a large scale.

## Figures and Tables

**Table 1:** Advantages and drawbacks of emerging extraction technologies.

Emerging Technology	Advantages	Drawbacks
USAE	↓ Energy, time and solvent consumption	Can induce oxidation pyrolysis
	↑ Solvent penetration into plant material (mixing effect) and extraction yields	Promote free radical formation
	Easy to use and low equipment cost	High ultrasound waves has deleterious effects on phytochemicals
	Facilitate mass transfer	↑ Temperature by cavitation
	Compatibility with GRAS solvents	Low selective
MWAE	↓ Time and solvent consumption	High energy consumption
	↑ Extraction yields	Excessive temperature (phytochemicals degradation)
	Cost-effective equipment	Oxidation reactions
	Easily to scale up	Low selective (large number of compounds extracted)
PEFAE	↓ Energy, time and solvent consumption	Very expensive equipment
	Very low changes in temperature	Need proper solvent and electrical conductivity
	Minimize degradation of thermolabile phytochemicals	
	Easily to scale up	
	High selectivity	
SFAE	↓Extraction time	High energy consumption
	No use toxic solvents	Very expensive and complex equipment
	Extracts are pure, and present high quality	Need co-solvent to ensure the correct extraction of polar compounds
	Effective (low viscosity and high diffusivity)	Scale up not feasible
	↑ Extraction yields	
	Continual process	
	Recycling supercritical fluid	
	Preserve thermolabile phytochemicals	
	Tunable supercritical fluid (solvent) density	

USAE: ultrasound-assisted extraction; MWAE: microwave-assisted extraction; PEFAE: pulsed electric field-assisted extraction; SFAE: supercritical fluid-assisted extraction. Information was obtained from previous studies [27,28,29,35,40,41].

**Table 2:** Effects of the application of encapsulated plant-based active ingredients in meat industry.

Plant Extracts	Concentration	Meat/Meat Product	Main Effects			Ref.
			Antioxidant Effects	Antimicrobial Effects	Other Effects	
Rosemary extract	800–1600 ppm	Beef meat	Reduce primary (peroxide values) and secondary (TBARs values) lipid oxidation	Inhibit the growth of microorganisms (total viable counts) during refrigerated storage	Minimum changes in color parameters	[118]
Orange essential oil and cactus acid fruit extract	0–5%	Emulsified meat system	Increase antioxidant activity (DPPH; ABTS) and reduce lipid oxidation during storage (TBARs)	NR	Increase fat content (with bioactive compounds from orange essential oil) and increase the total phenol content	[119]
Radish, hibiscus and beetroot extracts	0.4–7.29 g/kg	Cooked ham	NR	NR	Cooked ham with hibiscus presented the best color (instrumental and visual aspect parameters). From beetroot, the unencapsulated extract showed the best results	[120]
Lupulon–xanthohumol nanoliposome	50–200 ppm	Cooked beef sausage	Addition of liposome + nitrite successfully prevented lipid oxidation (TBARs)	Inhibit the growth of microorganisms (total viable counts and molds/yeast) (nitrite + nanoliposome combination presented the best results) during refrigerated storage. Nitrate + nanoliposome effectively inhibit the growth of *Clostridium perfringers* and coliforms	Liposome + nitrite successfully maintain the redness and did not produce changes in sensory properties of beef sausage (Customer acceptance)	[121]
*Allium sativum* essential oil	0.10%	Minced meat	NR	The essential oil microcapsules showed inhibitory effect (in essential oil concentration-dependent manner) against microorganisms growth (total aerobic mesophilic flora, sulfite-reducing anaerobes and *E. coli*)	NR	[122]
Thyme essential oil	1%	Hamburger-like meat products	NR	Inhibit the growth of thermotolerant coliforms and *E. coli*	NR	[123]
Prickly pear fruit extract	5%	Beef burger patties	NR	Samples treated with encapsulated prickly pear fruit extract showed lower values of mesophilic bacteria, *Enterobacteriaceae* and *Pseudomonas* spp.	Samples treated with encapsulated prickly pear fruit extract showed the smallest variations of color (redness) and texture. Maintain the pH values during storage, in contrast to control samples in which pH values increase progressively	[124]
Olive leaves extract	100 mg oleuropein / kg	Meat systems (with healthy oil mixture)	Higher oxidative stability (peroxide and TBARs values) than meat systems without extract (5 days under accelerated oxidative conditions). High antioxidant activity (FRAP and DPPH)	NR	Improvement of binding properties and texture	[125]
*Laurus nobilis* leaf extract	1000–1500 ppm	Minced beef	Inhibit oxidative degradation (peroxide and TBARs values)	Samples with extract presented the lowest values of total viable count and psychotropic count. Also inhibit the growth of *Staphylococcus aureus* and *E. coli*	Nanoencapsulated extracts reducing spoilage processes (lipolysis and non-protein volatile nitrogen). The score of sensory properties (general acceptance) decreased with the inclusion of extract, although all treatments had sensory ratings approved by the evaluators	[126]
Quinoa peptide-loaded nanoliposomes	3–5 mg/mL	Burger	Reduce primary (peroxide values) and secondary (TBARs values) lipid oxidation	Reduce the total bacterial count and growth of *S. aureus*, mold, and yeast	Reduce proteolytic activity derived from enzyme and/or microbial spoilage	[127]

NR: Effects not reported or studied.

## Data Availability

Not applicable.

## References

[1] Poore J., Nemecek T. (2018). Reducing food’s environmental impacts through producers and consumers. Science.

[2] Willett W., Rockström J., Loken B., Springmann M., Lang T., Vermeulen S., Garnett T., Tilman D., DeClerck F., Wood A. (2019). Food in the Anthropocene: The EAT–Lancet Commission on healthy diets from sustainable food systems. Lancet.

[3] Chen W., Ma S., Wang Q., McClements D.J., Liu X., Ngai T., Liu F. (2021). Fortification of edible films with bioactive agents: A review of their formation, properties, and application in food preservation. Crit. Rev. Food Sci. Nutr..

[4] McClements D.J., Bai L., Chung C. (2017). Recent Advances in the Utilization of Natural Emulsifiers to Form and Stabilize Emulsions. Annu. Rev. Food Sci. Technol..

[5] McClements D.J., Das A.K., Dhar P., Nanda P.K., Chatterjee N. (2021). Nanoemulsion-Based Technologies for Delivering Natural Plant-Based Antimicrobials in Foods. Front. Sustain. Food Syst..

[6] Domínguez R., Barba F.J., Gómez B., Putnik P., Bursać Kovačević D., Pateiro M., Santos E.M., Lorenzo J.M. (2018). Active packaging films with natural antioxidants to be used in meat industry: A review. Food Res. Int..

[7] Munekata P.E.S., Gullón B., Pateiro M., Tomasevic I., Domínguez R., Lorenzo J.M. (2020). Natural Antioxidants from Seeds and Their Application in Meat Products. Antioxidants.

[8] Pateiro M., Munekata P.E.S., Sant’Ana A.S., Domínguez R., Rodríguez-Lázaro D., Lorenzo J.M. (2021). Application of essential oils as antimicrobial agents against spoilage and pathogenic microorganisms in meat products. Int. J. Food Microbiol..

[9] Domínguez R., Munekata P.E.S., Pateiro M., Maggiolino A., Bohrer B., Lorenzo J.M. (2020). Red beetroot. A potential source of natural additives for the meat industry. Appl. Sci..

[10] Domínguez R., Gullón P., Pateiro M., Munekata P.E.S., Zhang W., Lorenzo J.M. (2020). Tomato as potential source of natural additives for meat industry. A review. Antioxidants.

[11] Domínguez R., Pateiro M., Gagaoua M., Barba F.J., Zhang W., Lorenzo J.M. (2019). A comprehensive review on lipid oxidation in meat and meat products. Antioxidants.

[12] Gómez B., Barba F.J., Domínguez R., Putnik P., Bursać Kovačević D., Pateiro M., Toldrá F., Lorenzo J.M. (2018). Microencapsulation of antioxidant compounds through innovative technologies and its specific application in meat processing. Trends Food Sci. Technol..

[13] Pateiro M., Gómez B., Munekata P.E.S., Barba F.J., Putnik P., Kovačević D.B., Lorenzo J.M. (2021). Nanoencapsulation of Promising Bioactive Compounds to Improve Their Absorption, Stability, Functionality and the Appearance of the Final Food Products. Molecules.

[14] Lorenzo J.M., Pateiro M., Domínguez R., Barba F.J., Putnik P., Kovačević D.B., Shpigelman A., Granato D., Franco D. (2018). Berries extracts as natural antioxidants in meat products: A review. Food Res. Int..

[15] Roselló-Soto E., Barba F.J., Lorenzo J.M., Dominguez R., Pateiro M., Mañes J., Moltó J.C. (2019). Evaluating the impact of supercritical-CO 2 pressure on the recovery and quality of oil from “horchata” by-products: Fatty acid profile, α-tocopherol, phenolic compounds, and lipid oxidation parameters. Food Res. Int..

[16] Pateiro M., Gómez-Salazar J.A., Jaime-Patlán M., Sosa-Morales M.E., Lorenzo J.M. (2021). Plant extracts obtained with green solvents as natural antioxidants in fresh meat products. Antioxidants.

[17] de Carvalho F.A.L., Munekata P.E.S., Lopes de Oliveira A., Pateiro M., Domínguez R., Trindade M.A., Lorenzo J.M. (2020). Turmeric (*Curcuma longa* L.) extract on oxidative stability, physicochemical and sensory properties of fresh lamb sausage with fat replacement by tiger nut (*Cyperus esculentus* L.) oil. Food Res. Int..

[18] Munekata P.E.S.E.S., Pateiro M., Zhang W., Dominguez R., Xing L., Fierro E.M., Lorenzo J.M. (2021). Health benefits, extraction and development of functional foods with curcuminoids. J. Funct. Foods.

[19] Pateiro M., Barba F.J., Domínguez R., Sant’Ana A.S., Mousavi Khaneghah A., Gavahian M., Gómez B., Lorenzo J.M. (2018). Essential oils as natural additives to prevent oxidation reactions in meat and meat products: A review. Food Res. Int..

[20] Munekata P.E.S., Pateiro M., Rodríguez-Lázaro D., Domínguez R., Zhong J., Lorenzo J.M. (2020). The Role of Essential Oils against Pathogenic Escherichia coli in Food Products. Microorganisms.

[21] Munekata P.E.S., Rocchetti G., Pateiro M., Lucini L., Domínguez R., Lorenzo J.M. (2020). Addition of plant extracts to meat and meat products to extend shelf-life and health-promoting attributes: An overview. Curr. Opin. Food Sci..

[22] Domínguez R., Zhang L., Rocchetti G., Lucini L., Pateiro M., Munekata P.E.S., Lorenzo J.M. (2020). Elderberry (*Sambucus nigra* L.) as potential source of antioxidants. Characterization, optimization of extraction parameters and bioactive properties. Food Chem..

[23] Vieira Teixeira da Silva D., dos Santos Baião D., de Oliveira Silva F., Alves G., Perrone D., Mere Del Aguila E., M Flosi Paschoalin V. (2019). Betanin, a Natural Food Additive: Stability, Bioavailability, Antioxidant and Preservative Ability Assessments. Molecules.

[24] Chhikara N., Kushwaha K., Sharma P., Gat Y., Panghal A. (2019). Bioactive compounds of beetroot and utilization in food processing industry: A critical review. Food Chem..

[25] Sigurdson G.T., Tang P., Giusti M.M. (2017). Natural colorants: Food colorants from natural sources. Annu. Rev. Food Sci. Technol..

[26] Belwal T., Chemat F., Venskutonis P.R., Cravotto G., Jaiswal D.K., Bhatt I.D., Devkota H.P., Luo Z. (2020). Recent advances in scaling-up of non-conventional extraction techniques: Learning from successes and failures. Trends Anal. Chem..

[27] Ameer K., Shahbaz H.M., Kwon J.-H.H. (2017). Green extraction methods for polyphenols from plant matrices and their byproducts: A review. Compr. Rev. Food Sci. Food Saf..

[28] Alirezalu K., Pateiro M., Yaghoubi M., Alirezalu A., Peighambardoust S.H., Lorenzo J.M. (2020). Phytochemical constituents, advanced extraction technologies and techno-functional properties of selected Mediterranean plants for use in meat products. A comprehensive review. Trends Food Sci. Technol..

[29] Reddy A.V.B., Moniruzzaman M., Madhavi V., Jaafar J. (2020). Recent improvements in the extraction, cleanup and quantification of bioactive flavonoids. Studies in Natural Products Chemistry.

[30] Cook C.M., Lanaras T. (2016). Essential Oils: Isolation, Production and Uses. Encyclopedia of Food and Health.

[31] Amirmohammadi F.Z., Azizi M., Nemati S.H., Iriti M., Vitalini S. (2020). Analysis of the essential oil composition of three cultivated Nepeta species from Iran. Z. fur Naturforsch. Sect. C J. Biosci..

[32] Andrade M., Ribeiro-Santos R., Silva A.S., Malik S. (2021). Essential Oils from Plants: Industrial Applications and Biotechnological Production. Exploring Plant Cells for the Production of Compounds of Interest.

[33] Baptiste Hzounda Fokou J., Michel Jazet Dongmo P., Fekam Boyom F., El-Shemy H. (2020). Essential Oil’s Chemical Composition and Pharmacological Properties. Essential Oils—Oils of Nature.

[34] Zin M.M., Anucha C.B., Bánvölgyi S. (2020). Recovery of Phytochemicals via Electromagnetic Irradiation (Microwave-Assisted-Extraction): Betalain and Phenolic Compounds in Perspective. Foods.

[35] Al Khawli F., Pateiro M., Domínguez R., Lorenzo J.M., Gullón P., Kousoulaki K., Ferrer E., Berrada H., Barba F.J. (2019). Innovative green technologies of intensification for valorization of seafood and their by-products. Mar. Drugs.

[36] Rente D., Paiva A., Duarte A.R. (2021). The role of hydrogen bond donor on the extraction of phenolic compounds from natural matrices using deep eutectic systems. Molecules.

[37] Chemat F., Rombaut N., Sicaire A.G., Meullemiestre A., Fabiano-Tixier A.S., Abert-Vian M. (2017). Ultrasound assisted extraction of food and natural products. Mechanisms, techniques, combinations, protocols and applications. A review. Ultrason. Sonochem..

[38] Bozinou E., Karageorgou I., Batra G., Dourtoglou V.G., Lalas S.I. (2019). Pulsed Electric Field Extraction and Antioxidant Activity Determination of Moringa oleifera Dry Leaves: A Comparative Study with Other Extraction Techniques. Beverages.

[39] Pacheco-Fernández I., Pino V. (2019). Extraction with ionic liquids-organic compounds. Liquid-Phase Extraction.

[40] Khaw K., Parat M., Shaw P.N., Falconer J.R. (2017). Solvent Supercritical Fluid Technologies to Extract Bioactive Compounds from Natural Sources: A Review. Molecules.

[41] Martínez J.M., Delso C., Álvarez I., Raso J. (2020). Pulsed electric field-assisted extraction of valuable compounds from microorganisms. Compr. Rev. Food Sci. Food Saf..

[42] Žlabur J.Š., Žutić I., Radman S., Pleša M., Brnčić M., Barba F.J., Rocchetti G., Lucini L., Lorenzo J.M., Domínguez R. (2020). Effect of different green extraction methods and solvents on bioactive components of chamomile (*Matricaria chamomilla* L.) flowers. Molecules.

[43] Žlabur J.Š., Brajer M., Voća S., Galić A., Radman S., Rimac-Brnčić S., Xia Q., Zhu Z., Grimi N., Barba F.J. (2021). Ultrasound as a promising tool for the green extraction of specialized metabolites from some culinary spices. Molecules.

[44] Jaimez-Ordaz J., Contreras-López E., Hernández-Sánchez T., González-Olivares L.G., Añorve-Morga J., Ramírez-Godínez J. (2021). Comparative evaluation of four extraction methods of antioxidant compounds from decatropis bicolor in aqueous medium applying response surface design. Molecules.

[45] Belwal T., Pandey A., Bhatt I.D., Rawal R.S. (2020). Optimized microwave assisted extraction (MAE) of alkaloids and polyphenols from Berberis roots using multiple-component analysis. Sci. Rep..

[46] Petrotos K., Giavasis I., Gerasopoulos K., Mitsagga C., Papaioannou C., Gkoutsidis P. (2021). Optimization of Vacuum-Microwave-Assisted Extraction of Natural Polyphenols and Flavonoids from Raw Solid Waste of the Orange Juice Producing Industry at Industrial Scale. Molecules.

[47] Barba F.J., Parniakov O., Pereira S.A., Wiktor A., Grimi N., Boussetta N., Saraiva J.A., Raso J., Martin-Belloso O., Witrowa-Rajchert D. (2015). Current applications and new opportunities for the use of pulsed electric fields in food science and industry. Food Res. Int..

[48] Nowacka M., Tappi S., Wiktor A., Rybak K., Miszczykowska A., Czyzewski J., Drozdzal K., Witrowa-Rajchert D., Tylewicz U. (2019). The Impact of Pulsed Electric Field on the Extraction of Bioactive Compounds from Beetroot. Foods.

[49] Moghaddam T.N., Elhamirad A.H., Saeidi Asl M.R., Shahidi Noghabi M. (2020). Pulsed electric field-assisted extraction of phenolic antioxidants from tropical almond red leaves. Chem. Pap..

[50] Ntourtoglou G., Tsapou E.A., Drosou F., Bozinou E., Lalas S., Tataridis P., Dourtoglou V. (2020). Pulsed Electric Field Extraction of α and β-Acids From Pellets of Humulus lupulus (Hop). Front. Bioeng. Biotechnol..

[51] Caballero A.S., Romero-García J.M., Castro E., Cardona C.A. (2020). Supercritical fluid extraction for enhancing polyphenolic compounds production from olive waste extracts. J. Chem. Technol. Biotechnol..

[52] Tyśkiewicz K., Konkol M., Rój E. (2018). The Application of Supercritical Fluid Extraction in Phenolic Compounds Isolation from Natural Plant Materials. Molecules.

[53] Wrona O., Rafińska K., Krakowska-Sieprawska A., Buszewski B. (2021). Comparative Studies of Selected Criteria Enabling Optimization of the Extraction of Polar Biologically Active Compounds from Alfalfa with Supercritical Carbon Dioxide. Molecules.

[54] Medina-Meza I.G., Boioli P., Barbosa-Cánovas G.V. (2016). Assessment of the Effects of Ultrasonics and Pulsed Electric Fields on Nutritional and Rheological Properties of Raspberry and Blueberry Purees. Food Bioprocess Technol..

[55] Tzima K., Brunton N.P., Lyng J.G., Frontuto D., Rai D.K. (2021). The effect of Pulsed Electric Field as a pre-treatment step in Ultrasound Assisted Extraction of phenolic compounds from fresh rosemary and thyme by-products. Innov. Food Sci. Emerg. Technol..

[56] Psarrou I., Oreopoulou A., Tsimogiannis D., Oreopoulou V. (2020). Extraction kinetics of phenolic antioxidants from the hydro distillation residues of rosemary and effect of pretreatment and extraction parameters. Molecules.

[57] Balachandran S., Kentish S.E., Mawson R., Ashokkumar M. (2006). Ultrasonic enhancement of the supercritical extraction from ginger. Ultrason. Sonochem..

[58] Da Porto C., Decorti D., Natolino A. (2016). Microwave pretreatment of Moringa oleifera seed: Effect on oil obtained by pilot-scale supercritical carbon dioxide extraction and Soxhlet apparatus. J. Supercrit. Fluids.

[59] Borrajo P., Pateiro M., Barba F.J., Mora L., Franco D., Toldrá F., Lorenzo J.M. (2019). Antioxidant and Antimicrobial Activity of Peptides Extracted from Meat By-products: A Review. Food Anal. Methods.

[60] Cao G., Prior R.L. (1998). Comparison of different analytical methods for assessing total antioxidant capacity of human serum. Clin. Chem..

[61] Amaral A.B., da Silva M.V., da Silva LANNES S.C. (2018). Lipid oxidation in meat: Mechanisms and protective factors—A review. Food Sci. Technol..

[62] Gulcin İ. (2020). Antioxidants and antioxidant methods: An updated overview. Arch. Toxicol..

[63] Alam M.N., Bristi N.J., Rafiquzzaman M. (2013). Review on in vivo and in vitro methods evaluation of antioxidant activity. Saudi Pharm. J..

[64] Martín J., Kuskoski E.M., Navas M.J., Asuero A.G., Justino G. (2017). Antioxidant Capacity of Anthocyanin Pigments. Flavonoids—From Biosynthesis to Human Health.

[65] Lorenzo J.M., Vargas F.C., Strozzi I., Pateiro M., Furtado M.M., Sant’Ana A.S., Rocchetti G., Barba F.J., Dominguez R., Lucini L. (2018). Influence of pitanga leaf extracts on lipid and protein oxidation of pork burger during shelf-life. Food Res. Int..

[66] Diwan R., Shinde A., Malpathak N. (2012). Phytochemical Composition and Antioxidant Potential of *Ruta graveolens* L. in vitro Culture Lines. J. Bot..

[67] Batool R., Khan M.R., Sajid M., Ali S., Zahra Z. (2019). Estimation of phytochemical constituents and in vitro antioxidant potencies of Brachychiton populneus (Schott & Endl.) R.Br. BMC Chem..

[68] Singleton V.L., Orthofer R., Lamuela-Raventós R.M., Lester Packer (1999). Analysis of total phenols and other oxidation substrates and antioxidants by means of folin-ciocalteu reagent. Methods in Enzymology.

[69] Czyżowska A., Siemianowska K., Śniadowska M., Nowak A. (2020). Bioactive compounds and microbial quality of stored fermented red beetroots and red beetroot juice. Pol. J. Food Nutr. Sci..

[70] Roriz C.L., Xavier V., Heleno S.A., Pinela J., Dias M.I., Calhelha R.C., Morales P., Ferreira I.C.F.R., Barros L. (2021). Chemical and Bioactive Features of *Amaranthus caudatus* L. Flowers and Optimized Ultrasound-Assisted Extraction of Betalains. Foods.

[71] Armesto J., Rocchetti G., Senizza B., Pateiro M., Barba F.J., Domínguez R., Lucini L., Lorenzo J.M. (2020). Nutritional characterization of *Butternut squash* (*Cucurbita moschata* D.): Effect of variety (Ariel vs. Pluto) and farming type (conventional vs. organic). Food Res. Int..

[72] López-Fernández O., Domínguez R., Pateiro M., Munekata P.E.S., Rocchetti G., Lorenzo J.M. (2020). Determination of polyphenols using liquid chromatography–tandem mass spectrometry technique (LC–MS/MS): A review. Antioxidants.

[73] Munekata P.E.S., Domínguez R., Franco D., Bermúdez R., Trindade M.A., Lorenzo J.M. (2017). Effect of natural antioxidants in Spanish salchichón elaborated with encapsulated n-3 long chain fatty acids in konjac glucomannan matrix. Meat Sci..

[74] Munekata P.E.S., Domínguez R., Campagnol P.C.B., Franco D., Trindade M.A., Lorenzo J.M. (2017). Effect of natural antioxidants on physicochemical properties and lipid stability of pork liver pâté manufactured with healthy oils during refrigerated storage. J. Food Sci. Technol..

[75] Corleto K.A., Singh J., Jayaprakasha G.K., Patil B.S. (2018). Storage Stability of Dietary Nitrate and Phenolic Compounds in Beetroot (*Beta vulgaris* ) and Arugula (*Eruca sativa* ) Juices. J. Food Sci..

[76] Escobar-Avello D., Mardones C., Saéz V., Riquelme S., von Baer D., Lamuela-Raventós R.M., Vallverdú-Queralt A. (2021). Pilot-plant scale extraction of phenolic compounds from grape canes: Comprehensive characterization by LC-ESI-LTQ-Orbitrap-MS. Food Res. Int..

[77] Pateiro M., Vargas F.C., Chincha A.A.I.A., Sant’Ana A.S., Strozzi I., Rocchetti G., Barba F.J., Domínguez R., Lucini L., do Amaral Sobral P.J. (2018). Guarana seed extracts as a useful strategy to extend the shelf life of pork patties: UHPLC-ESI/QTOF phenolic profile and impact on microbial inactivation, lipid and protein oxidation and antioxidant capacity. Food Res. Int..

[78] Ed-Dra A., Filali F.R., Lo Presti V., Zekkori B., Nalbone L., Bouymajane A., Trabelsi N., Lamberta F., Bentayeb A., Giuffrida A. (2020). Chemical composition, antioxidant capacity and antibacterial action of five Moroccan essential oils against *Listeria monocytogenes* and different serotypes of *Salmonella enterica*. Microb. Pathog..

[79] Ghasemi G., Alirezalu A., Ghosta Y., Jarrahi A., Safavi S.A., Abbas-Mohammadi M., Barba F.J., Munekata P.E.S., Domínguez R., Lorenzo J.M. (2020). Composition, antifungal, phytotoxic, and insecticidal activities of thymus kotschyanus essential oil. Molecules.

[80] Fan S., Chang J., Zong Y., Hu G., Jia J. (2018). GC-MS analysis of the composition of the essential oil from *Dendranthema indicum* Var. Aromaticum using three extraction methods and two columns. Molecules.

[81] Ozaki M.M., dos Santos M., Ribeiro W.O., de Azambuja Ferreira N.C., Picone C.S.F., Domínguez R., Lorenzo J.M., Pollonio M.A.R. (2021). Radish powder and oregano essential oil as nitrite substitutes in fermented cooked sausages. Food Res. Int..

[82] McClements D.J. (2018). Encapsulation, protection, and delivery of bioactive proteins and peptides using nanoparticle and microparticle systems: A review. Adv. Colloid Interface Sci..

[83] McClements D.J. (2020). Nano-enabled personalized nutrition: Developing multicomponent-bioactive colloidal delivery systems. Adv. Colloid Interface Sci..

[84] Kharat M., McClements D.J. (2019). Recent advances in colloidal delivery systems for nutraceuticals: A case study—Delivery by Design of curcumin. J. Colloid Interface Sci..

[85] McClements D.J., Li F., Xiao H. (2015). The nutraceutical bioavailability classification scheme: Classifying nutraceuticals according to factors limiting their oral bioavailability. Annu. Rev. Food Sci. Technol..

[86] McClements D.J., McClements D.J. (2014). Nanoparticle- and Microparticle-Based Delivery Systems: Encapsulation, Protection and Release of Active Compounds.

[87] McClements D.J. (2018). Delivery by Design (DbD): A Standardized Approach to the Development of Efficacious Nanoparticle- and Microparticle-Based Delivery Systems. Compr. Rev. Food Sci. Food Saf..

[88] Flanagan J., Singh H. (2006). Microemulsions: A potential delivery system for bioactives in food. Crit. Rev. Food Sci. Nutr..

[89] Garti N., Aserin A., Garti N., McClements D.J. (2012). Micelles and microemulsions as food ingredient and nutraceutical delivery systems. Encapsulation Technologies and Delivery Systems for Food Ingredients and Nutraceuticals.

[90] Gupta S. (2011). Biocompatible microemulsion systems for drug encapsulation and delivery. Curr. Sci..

[91] Narang A.S., Delmarre D., Gao D. (2007). Stable drug encapsulation in micelles and microemulsions. Int. J. Pharm..

[92] McClements D.J. (2012). Nanoemulsions versus microemulsions: Terminology, differences, and similarities. Soft Matter.

[93] Deshpande P.P., Biswas S., Torchilin V.P. (2013). Current trends in the use of liposomes for tumor targeting. Nanomedicine.

[94] Maherani B., Arab-Tehrany E., Mozafari M.R., Gaiani C., Linder M. (2011). Liposomes: A Review of Manufacturing Techniques and Targeting Strategies. Curr. Nanosci..

[95] Malam Y., Loizidou M., Seifalian A.M. (2009). Liposomes and nanoparticles: Nanosized vehicles for drug delivery in cancer. Trends Pharmacol. Sci..

[96] Mozafari M.R., Johnson C., Hatziantoniou S., Demetzos C. (2008). Nanoliposomes and their applications in food nanotechnology. J. Liposome Res..

[97] Sawant R.R., Torchilin V.P. (2010). Liposomes as’smart’ pharmaceutical nanocarriers. Soft Matter.

[98] Taylor T.M., Davidson P.M., Bruce B.D., Weiss J. (2005). Liposomal nanocapsules in food science and agriculture. Crit. Rev. Food Sci. Nutr..

[99] McClements D.J. (2011). Edible nanoemulsions: Fabrication, properties, and functional performance. Soft Matter.

[100] McClements D.J., Rao J. (2011). Food-Grade nanoemulsions: Formulation, fabrication, properties, performance, Biological fate, and Potential Toxicity. Crit. Rev. Food Sci. Nutr..

[101] Kesisoglou F., Panmai S., Wu Y. (2007). Nanosizing—Oral formulation development and biopharmaceutical evaluation. Adv. Drug Deliv. Rev..

[102] Pardeike J., Hommoss A., Müller R.H. (2009). Lipid nanoparticles (SLN, NLC) in cosmetic and pharmaceutical dermal products. Int. J. Pharm..

[103] Helgason T., Awad T.S., Kristbergsson K., McClements D.J., Weiss J. (2009). Effect of surfactant surface coverage on formation of solid lipid nanoparticles (SLN). J. Colloid Interface Sci..

[104] McClements D.J. (2017). Recent progress in hydrogel delivery systems for improving nutraceutical bioavailability. Food Hydrocoll..

[105] Oh J.K., Drumright R., Siegwart D.J., Matyjaszewski K. (2008). The development of microgels/nanogels for drug delivery applications. Prog. Polym. Sci..

[106] Joye I.J., McClements D.J. (2014). Biopolymer-based nanoparticles and microparticles: Fabrication, characterization, and application. Curr. Opin. Colloid Interface Sci..

[107] dos Santos C., Buera P., Mazzobre F. (2017). Novel trends in cyclodextrins encapsulation. Applications in food science. Curr. Opin. Food Sci..

[108] Kfoury M., Hădărugă N.G., Hădărugă D.I., Fourmentin S. (2016). Cyclodextrins as encapsulation material for flavors and aroma. Encapsulations.

[109] Kfoury M., Auezova L., Greige-Gerges H., Fourmentin S. (2019). Encapsulation in cyclodextrins to widen the applications of essential oils. Environ. Chem. Lett..

[110] Munekata P.E.S., Pateiro M., Bellucci E.R.B., Domínguez R., da Silva Barretto A.C., Lorenzo J.M. (2021). Strategies to increase the shelf life of meat and meat products with phenolic compounds. Advances in Food and Nutrition Research.

[111] Pateiro M., Domínguez R., Bermúdez R., Munekata P.E.S., Zhang W., Gagaoua M., Lorenzo J.M. (2019). Antioxidant active packaging systems to extend the shelf life of sliced cooked ham. Curr. Res. Food Sci..

[112] Kostić A., Milinčić D.D., Barać M.B., Shariati M.A., Tešić Ž.L., Pešić M.B. (2020). The application of pollen as a functional food and feed ingredient—The present and perspectives. Biomolecules.

[113] de Florio Almeida J., dos Reis A.S., Heldt L.F.S., Pereira D., Bianchin M., de Moura C., Plata-Oviedo M.V., Haminiuk C.W.I., Ribeiro I.S., da Luz C.F.P. (2017). Lyophilized bee pollen extract: A natural antioxidant source to prevent lipid oxidation in refrigerated sausages. LWT-Food Sci. Technol..

[114] Turhan S., Saricaoglu F.T., Mortas M., Yazici F., Genccelep H. (2017). Evaluation of Color, Lipid Oxidation and Microbial Quality in Meatballs Formulated with Bee Pollen During Frozen Storage. J. Food Process. Preserv..

[115] Turhan S., Yazici F., Saricaoglu T., Mortas M., Genccelep H. (2014). Evaluation of the nutritional and storage quality of meatballs formulated with bee pollen. Korean J. Food Sci. Anim. Resour..

[116] Vargas-Sánchez R.D., Torrescano-Urrutia G.R., Acedo-Félix E., Carvajal-Millán E., González-Córdova A.F., Vallejo-Galland B., Torres-Llanez M.J., Sánchez-Escalante A. (2014). Antioxidant and antimicrobial activity of commercial propolis extract in beef patties. J. Food Sci..

[117] Vargas-Sánchez R.D., Torrescano-Urrutia G.R., Torres-Martínez B.D.M., Pateiro M., Lorenzo J.M., Sánchez-Escalante A. (2019). Propolis extract as antioxidant to improve oxidative stability of fresh patties during refrigerated storage. Foods.

[118] Abandansarie S.S.R., Ariaii P., Charmchian Langerodi M. (2019). Effects of encapsulated rosemary extract on oxidative and microbiological stability of beef meat during refrigerated storage. Food Sci. Nutr..

[119] Almaráz-Buendia I., Hernández-Escalona A., González-Tenorio R., Santos-Ordoñez N., Espino-García J.J., Martínez-Juárez V., Meza-Nieto M.A., Campos Montiel R.G. (2019). Producing an Emulsified Meat System by Partially Substituting Pig Fat with Nanoemulsions that Contain Antioxidant Compounds: The Effect on Oxidative Stability, Nutritional Contribution, and Texture Profile. Foods.

[120] Dias S., Castanheira E.M.S., Fortes A.G., Pereira D.M., Rodrigues A.R.O., Pereira R., Gonçalves M.S.T. (2020). Application of Natural Pigments in Ordinary Cooked Ham. Molecules.

[121] Khatib N., Varidi M.M.J., Mohebbi M., Varidi M.M.J., Hosseini S.M.H. (2020). Replacement of nitrite with lupulon–xanthohumol loaded nanoliposome in cooked beef-sausage: Experimental and model based study. J. Food Sci. Technol..

[122] Najjaa H., Chekki R., Elfalleh W., Tlili H., Jaballah S., Bouzouita N. (2020). Freeze-dried, oven-dried, and microencapsulation of essential oil from Allium sativum as potential preservative agents of minced meat. Food Sci. Nutr..

[123] Radünz M., dos Santos Hackbart H.C., Camargo T.M., Nunes C.F.P., de Barros F.A.P., Dal Magro J., Filho P.J.S., Gandra E.A., Radünz A.L., da Rosa Zavareze E. (2020). Antimicrobial potential of spray drying encapsulated thyme (*Thymus vulgaris*) essential oil on the conservation of hamburger-like meat products. Int. J. Food Microbiol..

[124] Parafati L., Palmeri R., Trippa D., Restuccia C., Fallico B. (2019). Quality Maintenance of Beef Burger Patties by Direct Addiction or Encapsulation of a Prickly Pear Fruit Extract. Front. Microbiol..

[125] Robert P., Zamorano M., González E., Silva-Weiss A., Cofrades S., Giménez B. (2019). Double emulsions with olive leaves extract as fat replacers in meat systems with high oxidative stability. Food Res. Int..

[126] Tometri S.S., Ahmady M., Ariaii P., Soltani M.S. (2020). Extraction and encapsulation of Laurus nobilis leaf extract with nano-liposome and its effect on oxidative, microbial, bacterial and sensory properties of minced beef. J. Food Meas. Charact..

[127] Yekta M.M., Rezaei M., Nouri L., Azizi M.H., Jabbari M., Eş I., Khaneghah A.M. (2020). Antimicrobial and antioxidant properties of burgers with quinoa peptide-loaded nanoliposomes. J. Food Saf..

